# A hybrid sub-lineage of *Listeria monocytogenes* comprising hypervirulent isolates

**DOI:** 10.1038/s41467-019-12072-1

**Published:** 2019-09-30

**Authors:** Yuelan Yin, Hao Yao, Swapnil Doijad, Suwei Kong, Yang Shen, Xuexue Cai, Weijun Tan, Yuting Wang, Youwei Feng, Zhiting Ling, Guoliang Wang, Yachen Hu, Kai Lian, Xinyu Sun, Yuliang Liu, Chuanbin Wang, Kuhua Jiao, Guoping Liu, Ruilong Song, Xiang Chen, Zhiming Pan, Martin J. Loessner, Trinad Chakraborty, Xin’an Jiao

**Affiliations:** 1 Jiangsu Key Laboratory of Zoonosis, Key Laboratory of Prevention and Control of Biological Hazard Factors (Animal Origin) for Agrifood Safety and Quality, Ministry of Agriculture and Rural Affairs of the People’s Republic of China, Yangzhou University, 48 East Wenhui Road, Yangzhou, 225009 Jiangsu Province China; 2 Jiangsu Co-Innovation Center for Prevention and Control of Important Animal Infectious Disease and Zoonoses, 48 East Wenhui Road, Yangzhou, 225009 Jiangsu Province China; 3 Institute of Medical Microbiology, Justus-Liebig University, Giessen, 35394 Germany; 4 German Center for Infection Research (DZIF), Partner Site Gießen-Marburg-Langen, Campus Gießen, Justus-Liebig University, Gießen, 35394 Germany; 5 Laboratory of Food Microbiology, Institute of Food, Nutrition and Health, ETH Zurich, 8092 Zurich, Switzerland; 6 China Animal Disease Control Center, No.17 Tiangui Street, Daxing District, 102618 Beijing, China; 7 Xuyi Center for Animal Disease Control and Prevention, Xuyi City, Jiangsu Province China

**Keywords:** Bacterial evolution, Bacterial genomics, Pathogens

## Abstract

The foodborne pathogen *Listeria monocytogenes* (Lm) is a highly heterogeneous species and currently comprises of 4 evolutionarily distinct lineages. Here, we characterize isolates from severe ovine listeriosis outbreaks that represent a hybrid sub-lineage of the major lineage II (HSL-II) and serotype 4h. HSL-II isolates are highly virulent and exhibit higher organ colonization capacities than well-characterized hypervirulent strains of Lm in an orogastric mouse infection model. The isolates harbour both the Lm Pathogenicity Island (LIPI)-1 and a truncated LIPI-2 locus, encoding sphingomyelinase (SmcL), a virulence factor required for invasion and bacterial translocation from the gut, and other non-contiguous chromosomal segments from another pathogenic species, *L*. *ivanovii*. HSL-II isolates exhibit a unique wall teichoic acid (WTA) structure essential for resistance to antimicrobial peptides, bacterial invasion and virulence. The discovery of isolates harbouring pan-species virulence genes of the genus *Listeria* warrants global efforts to identify further hypervirulent lineages of Lm.

## Abstract (web summary)

*Listeria monocytogenes* isolates are highly heterogeneous and exhibit different levels of virulence. Here, the authors identify hypervirulent isolates that represent a hybrid sub-lineage of the major lineage II harbouring virulence factors from *Listeria ivanovii* and wall teichoic acids found in major lineage I.

## Introduction

L*isteria monocytogenes* (Lm) is an important foodborne pathogen and a major cause of deaths associated with bacterial foodborne infections in the Western world^1^. It is a Gram-positive intracellular pathogen causing meningitis and sepsis particularly among the newborn, elderly, and immunocompromised individuals^2^. An association between contaminated food in both epidemic and sporadic infections is well-documented^1^, and it is now recognized that the long incubation period of invasive listeriosis can make source-distribution associations difficult^3^. Whole-genome sequencing (WGS) is increasingly used as the basis for developing genotyping methods to demonstrate occurrence and pinpoint circulation of strains with great precision^4^. Recent outbreaks of severe invasive listeriosis in South Africa, Europe, and Australia with serious social and economic implications underline the importance of coupling food control measures with surveillance and epidemiology^3,5,6^.

Lm is a highly heterogeneous species that currently classifies into four evolutionary lineages, thirteen serotypes, and four PCR serogroups^7,8^. Multi-locus sequence-based typing sub-categorize distinct clonal complexes (CC) within lineages i.e., sublineages (SL), that are associated with severe disease manifestations, such as invasive listeriosis^9^. National surveillance data from France and the Netherlands indicate the predominance of highly invasive CC1, CC2, CC3, CC4, and more recently CC6 isolates in systemic infections in humans with neurological forms of listeriosis^9,10^.

The origin of heterogeneity of virulence in Lm isolates has been studied in detail. Genomic traits associated with virulence include the Listeria Pathogenicity Island (LIPI)-1, that carries the master regulator of virulence transcription *prfA*, and a cluster of virulence genes required for escape from vacuolar compartments (*hly* and *plcA*), actin-based motility (*actA*), and cell-to-cell spread (*mpl*, *plcB*, and *orfX*)^2^. Additional sub-lineage-specific pathogenicity islands, such as LIPI-3 and LIPI-4, that encode the bacteriocin LLS and a putative cellobiose-family phosphotransferase system, respectively, promote when present in combination with LIPI-1 bacterial colonization, and enhance neurovirulence^2^. Other genes clearly associated with human disease include the presence of full-length internalin A as well as gene clusters required for teichoic acid biosynthesis in lineage I Serovar (Sv) 4b strains^2^. A further pathogenicity island LIPI-2, encoding a number of internalins and the enzyme sphingomyelinase, is specific for a separate pathogenic species, *L*. *ivanovii*^11^.

Here, we describe a group of rhamnose-negative *L*. *monocytogenes* isolates obtained over a period of 5 years from different sources that harbor distinct genes from the species *L*. *ivanovii*. They cannot be typed by the widely used PCR-based serogrouping scheme of Doumith et al.^7^, and represent founding members of the serotype 4h. In particular, these isolates are highly virulent in orogastric models of infection in rodents (mice and guinea pigs). We designate these isolates as members of the hybrid sub-lineage II (HSL-II). Here, we examine genomic features of these isolates through comparative analysis with those of other lineages and integrate this information with experimental data to explore the basis of its hypervirulent phenotype.

## Results

### Atypical *L*. *monocytogenes* isolates as causative agents of high mortality

The isolates NTSN, XYSN, and 15LG were obtained from separate listeriosis outbreaks on goat farms in 2011, 2012, and 2015, located in a remote region of Jiangsu Province, China. An outbreak with the NTSN, a lineage I serotype 4b strain, resulted in the death of 18 animals in a flock of ~5000 animals. In the case of XYSN (18%; 36/200) and 15LG (3%; 5/150), goats succumbed to infection. For the 2015 outbreak, rapid identification of isolates allowed treatment of the animals with cephalexin and ampicillin thus reducing the number of deaths recorded. Sickened goats were diagnosed with meningitis, exhibited aberrant gait and flock behavior with severe neurological symptoms manifested as a tendency to circle in one direction when walking. The head tilt was accompanied with uncoordinated movement and an inability to eat and drink. At the stage of impending death, goats were prostrate with viscid, meliceral, saliva flowing from the mouth. In all cases, severe gross pathological changes during autopsy were observed in the brain of the goats, including meningeal congestion. Culturing of brain tissue samples onto CHROMagar^TM^ plates grew colonies with a blue/green sheen and opaque halos. Phenotypic tests performed revealed characteristics typical of *L*. *monocytogenes*. Cultures visualized as Gram-positive rods that were, catalase-positive and oxidase-negative, and unable to utilize D-mannitol and D-xylose, but fermented α-methyl D-mannoside. Atypically, these isolates did not produce acid from L-rhamnose, a feature that is characteristic of the species *L*. *monocytogenes*. Colonies were hemolytic on blood agar plates but surprisingly also tested positive in the Christie–Atkins–Munch-Petersen (CAMP) reaction using *Rhodococcus equi*. These isolates were untypable by the PCR-based serogroup assay^7^. Agglutination studies with antisera to somatic antigens indicated that they belonged to the serogroup 4. All isolates reacted with antisera for flagellar H-A and H-B antigen but not with H-C antigen, indicating that they are members of a different serovar that we now designate as 4h (Supplementary Table 5). The antibiotic-resistance phenotypic profile showed that they were sensitive to most antibiotics, except for an intermediate resistance to clindamycin (Supplementary Table 6). A single rhamnose-negative *L*. *monocytogenes* environmental isolate (16E) displaying the above characteristics was also isolated in 2016 from goat-rearing farm. We selected three independent representative isolates XYSN, 15LG, and 16E obtained from the outbreaks and the environment, for whole-genome-based sequence analysis. Experimental studies were carried out with XYSN, a representative isolate obtained from the earliest outbreak of 2011.

### *L*.* monocytogenes* isolates XYSN, 15LG, and 16E are hypervirulent

**Fig. 1:**
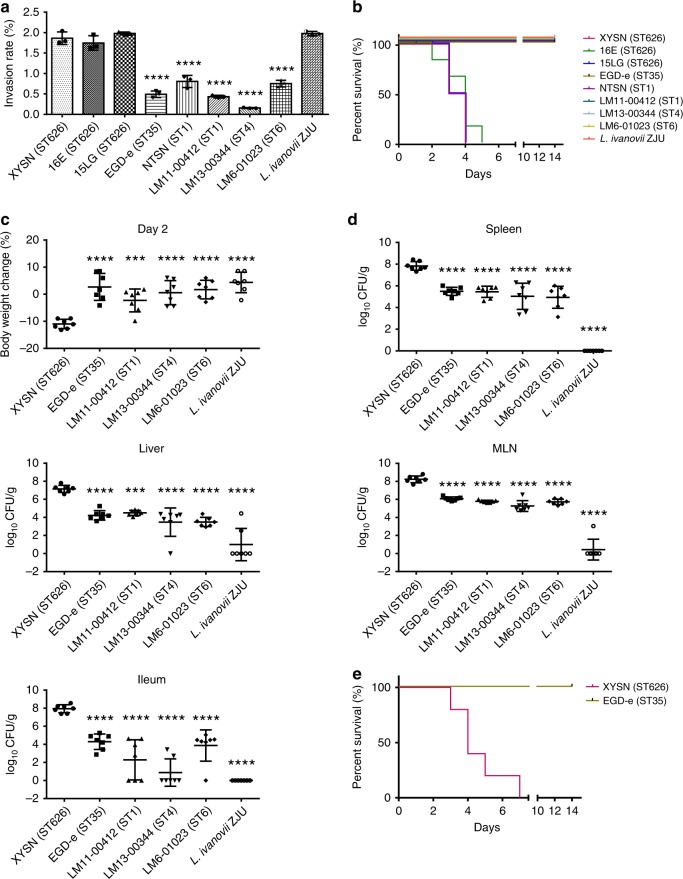
Comparative analysis of virulence properties of lineage I and II strains with XYSN, 15LG, and 16E. **a** Invasion capacities of XYSN, 15LG, 16E, EGD-e, NTSN, LM11-00412, LM13-00344, LM6-01023, and *L*. *ivanovii* ZJU were examined using the Caco-2 BBe cell line (MOI = 20, invasion for 15 min). Bacterial invasion rate = (number of cells internalized/number of initial bacteria in wells) × 100%. Error bars represent SD, *n* = 3 independent experiments. Statistical analyses were carried out by Dunnett's multiple comparisons test: ****P < 0.0001. **b** Survival curves of C57BL/6 mice (*n* = 6/group) were determined via orogastric inoculation of 2 × 10^8^ CFU of XYSN, 15LG, 16E, EGD-e, NTSN, LM11-00412, LM13-00344, LM6-01023, and *L*. *ivanovii* ZJU. The data represent two independent experiments. **c** Changes in body weights were recorded on day 2 post infection with 2 × 10^8^ CFU of XYSN, EGD-e, LM11-00412, LM13-00344, LM6-01023, and *L*. *ivanovii* ZJU. **d** Organs colonization propensities of listerial strains. Strains were inoculated at a dose of 2 × 10^8^ CFU. The data presented are at 48 h post infection. The log CFUs/g represents the mean of seven mice per group. Each dot represents an organ from one infected mouse. Error bars represent SD, *n* = 3 independent experiments. Statistical analyses were carried out by Dunnett's multiple comparisons test: ***P < 0.001, ****P < 0.0001. **e** Survival curves of guinea pigs (*n* = 10/group) inoculated intragastrically with 1 × 10^9^ CFU of XYSN and EGD-e, respectively, and observed continuously for 14 days. The data represent two independent experiments

We examined the abilities of the isolates XYSN, 15LG, and 16E to invade the human intestinal epithelial Caco-2 BBe cell line. We compared this to the invasive abilities of reference hypervirulent strains, LM11-00412 (Sv4b; Lineage I; CC1), LM13-00344 (Sv4b; Lineage I; CC4), LM6-01023 (Sv4b; Lineage I; CC6) together with other well-characterized strains, i.e., EGD-e (Sv1/2a; Lineage II; CC9), NTSN (Sv4b; Lineage I; CC1), and *L*. *ivanovii* ZJU (Sv5). Invasion rates of XYSN, 15LG, and 16E were similar, and significantly higher than all other strains except *L*. *ivanovii* ZJU (Fig. 1a), indicating that an enhanced ability to invade human intestinal cells is a defining characteristic of these isolates.

We assessed the virulence potential of XYSN, 15LG, and 16E relative to reference strains in a mouse model of listeriosis. C57BL/6 mice orogastrically inoculated with isolates XYSN, 15LG, and 16E at a dose of 2 × 10^8^ CFU, progressively succumbed to infection, from between days 2 and 5 (Fig. 1b): no deaths were recorded for any of the other Lm isolates or *L*. *ivanovii* ZJU tested using the same dose (Fig. 1b).

We next examined for the organ colonization abilities of XYSN, *L*. *ivanovii* ZJU, EGD-e, and the reference CC1, CC4, and CC6 strains. For these experiments, all strains were o.g. inoculated at a dose of 2 × 10^8^ CFU. At 2 days post infection, bacterial loads in the ileum, MLN, spleen, as well as liver in XYSN-infected group were significantly higher than both EGD-e and the three strains that are representatives of the hypervirulent CC1, CC4, and CC6 clones^9^. The numbers of bacteria detected in individual MLN (1/7) and livers (2/7) in *L*. *ivanovii* ZJU-infected group were significantly lower than that of all other strains tested here (Fig. 1d). XYSN-infected mice exhibited a significantly higher loss of body weight as compared with infected mice of the other five groups (Fig. 1c). The ability of XYSN to colonize all four organs on day 5 post infection was also higher than that of all the other strains tested, even though the infection dose of XYSN was 100-fold lower (2 × 10^6^ vs 2 × 10^8^ CFU), as it was lethal when administered at higher doses (Supplementary Fig. 1).

We further examined the virulence potential of XYSN by comparing it with strain EGD-e in an orogastric guinea pig infection model. All of the infected guinea pigs succumbed when exposed to an orogastric dose of 1 × 10^9^ CFU of XYSN at day 7, with no deaths recorded when using the EGD-e strain (Fig. 1e).

### Discovery of the hybrid sub-lineage HSL-II

We performed WGS of XYSN, 15LG, and 16E to understand the genetic basis of hypervirulence as well as to resolve events underlying the discrepant phenotypes viz., rhamnose-negative, sero-untypability, and the CAMP phenomenon, seen with these isolates.

The genome sizes of XYSN, 15LG, and 16E were similar at ~2.99 Mb, and exhibited an average nucleotide identity (ANI) of >99.5% to each other. The ANIb to the type strain NCTC 10357 was 97.01%, and the in silico DNA–DNA hybridization (*is*DDH) values were around 76.20% [73.2–78.9%], indicating that they are indeed bona fide members of the species *L*. *monocytogenes*. MLST analysis revealed these isolates are members of a hitherto unidentified sequence type (ST) and clonal complex (CC). These isolates have been assigned as ST626 and CC33 by the *Listeria* MLST Pasteur database, and are the only isolates in either category in the database.

**Fig. 2:**
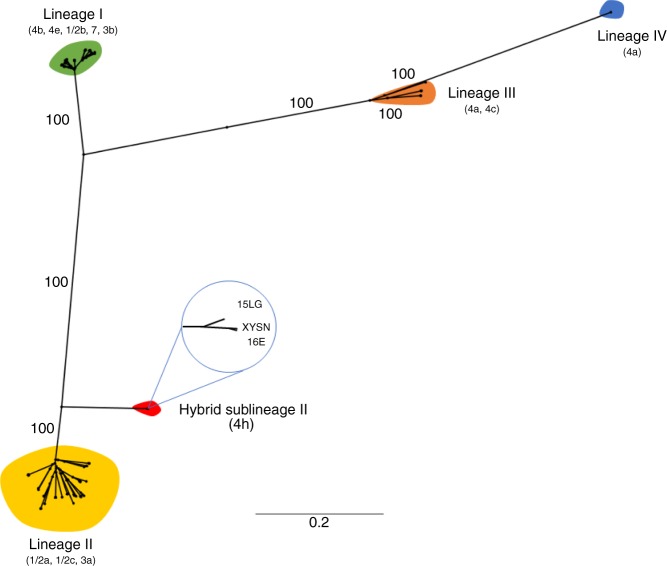
Comparative phylogenomic analysis of *L*. *monocytogenes*. A phylogenomic tree based on the comparative analysis of the core genes derived from the closed genomes of 144 *L*. *monocytogenes* strains together with XYSN, 15LG, and 16E. The maximum likelihood phylogeny was inferred using RAxML (with 100 bootstrap values) based on the concatenated core gene sequence (*n* = 1977 CDS, 1.69 Mb). *L*. *monocytogenes* XYSN, 15LG, and 16E formed a separate highly supported clade (100). Representative serotypes within the lineages are shown in parentheses

The phylogenomic relationship of XYSN, 15LG, and 16E was compared with the closed genomes of a representative set of 144 strains covering all of the currently known four lineages. Phylogenomic analysis based on a concatenated set of core genes (*n* = 1977, 1.69 Mb) distributed these 144 strains into the four known lineages, a topology that is identical to that reported previously^8,12–14^. XYSN, 15LG, and 16E clustered indistinguishably into a single clade, forming a discrete unit that is distinct from clades that represent the currently known four lineages (Fig. 2). Based on the characteristics describe above, we designate these isolates as members of a hybrid sub-lineage II (HSL-II).

### Genome features of hybrid sub-lineage II isolates

**Fig. 3:**
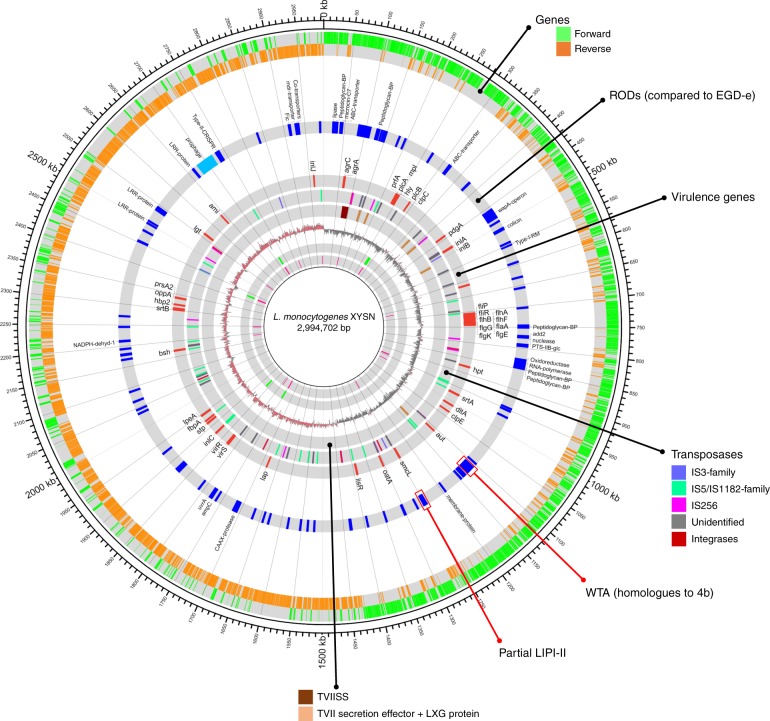
Genomic features of *L*. *monocytogenes* HSL-II isolate XYSN. The image depicts the closed genome of the *L*. *monocytogenes* HSL-II isolate XYSN. The innermost to outermost (gray) circles indicate the respective locations of tRNA, rRNA, GC-skew, T7SS, transposases, virulence genes, chromosomal region of differences (ROD) in XYSN compared with the EGD-e, forward–reverse genes, and an ideogram indexing base pairs. The figure was constructed using Circos v0.69

The genomes of HSL-II isolates harbor the Listeria Pathogenicity Island (LIPI)-1 encoding the *hly* gene required for production of the hemolytic toxin listeriolysin LLO, which is defining property for the species Lm. Sub-lineage-specific pathogenicity islands i.e., LIPI-3 and LIPI-4, encoding the bacteriocin LLS and a putative cellobiose-family phosphotransferase system, respectively, were absent in the HSL-II genomes. The LIPI-2 locus that encodes for the sphingomyelinase *smcL* and the internalins i-*inlF* and i-*inlE* is exclusive to the species *L*. *ivanovii*. Remarkably, a truncated LIPI-2 was detected in the genomes of HSL-II isolates (Supplementary Fig. 2). Also, many other genomic traits, e.g., the internalins (*inlA*, *inlB*, *inlC*, *inlJ*, and *inlP*), the invasion-associated protein (*iap*), and regulators (*vir*RS) that are associated with virulence in many other Lm lineages, are also present in these isolates (Fig. 3; Supplementary Table 7).

Compared with XYSN, 15LG and 16E differed by 4165 and 42 SNVs in their core genome, and harbored additional sets of 98 and 97 genes, respectively. Many of these additional genes are associated with metabolic activities for e.g., utilization of glucosides such as arbutin- and salicin-phosphotransferase systems, metabolism of amino acid such as arginine and proline, and a ESAT-6-like secretion system (ESS) (Supplementary Table 8). A 39,213 bp prophage was absent in 15LG. This data showed 15LG and 16E were closely related to XYSN and suggested their origin from a recent common ancestor.

### Comparative genomic analysis reveals unique evolutionary path of XYSN

Based on the relatively close phylogenomic association between the XYSN and EGD-e, we carried out a detailed comparative analysis between the genomes of both strains. The core chromosomal content varied by a total of 54,371 SNPs and indicated the presence of 78 regions of difference (ROD) (Fig. 3). The dN/dS ratio of coding-region-SNPs was 0.37, while Ts/Tv (transition to transversion) ratio was 2.06 indicating evolution through long-term negative or purifying selection process. Of the 78 RODs observed, 31 encoded functional genes, while the remaining 47 were associated with one or more transposases. A homology search of genes in the 31 RODs with the NCBI nucleotide database revealed that eight of the nine regions that had hitherto not been observed in *L*. *monocytogenes* were *L*. *ivanovii*-specific (Supplementary Table 9). These included the truncated LIPI-2 described above, a *ugp* operon involved in uptake of sn-glycerol-3-phosphate, genes for menaquinone biosynthesis, and a cobalt transporter operon. Two regions, one encoding a putative type seven secretion effector protein system (T7SS) followed by polymorphic toxin harboring LxG domain protein, and a second region encoding for a toxin-immunity protein module tRNA-nuclease WapA, were exclusively present in XYSN. The remaining 22 RODs encoded genes present in isolates from other lineages of *L*. *monocytogenes*, as well as in other *Listeria* species. They include a gene cluster associated with the modification of cell-wall teichoic acid (XYSN-WTA), peptidoglycan-binding proteins, a putative lipase and oxidoreductase, ABC transporters, Type II CRISPR-Cas systems, leucine-rich cell surface proteins, a type I restriction modification system, and protein harboring a Fic-domain, as well as several other hypothetical proteins (see Fig. 3).

A highly unusual feature of the XYSN genome was the presence of a large (*n* = 90) number of transposase genes. Of these, 28 could be classified as members of IS5/IS1182-, 16 of the IS3-, and 15 of IS256 families. All other putative transposases harbored a characteristic helix-turn-helix (HTH) family motif required for binding to DNA (Supplementary Table 10). Only two of these IS3-related transposases, designated as ISLmo1 insertion elements, were previously reported to be present in the *L*. *monocytogenes* chromosome^15^. The majority of these transposases were closely associated with the RODs, suggesting a role in generating genome plasticity.

### A gene cluster associated with wall teichoic acid modification promotes bacterial invasion

As the enhanced invasive potential of XYSN for intestinal epithelial cells is associated with its hypervirulent phenotype, we used genome-wide saturating transposon mutagenesis to screen for those mutants that were defective for invasion of the Caco-2 BBe cell line (Supplementary Fig. 3). From this analysis, three mutants, that consistently exhibited 20-fold reduced invasive capacity, were examined in further detail. The transposon insertion sites in these three mutants were determined and mapped to the loci LMxysn_0462, LMxysn_1095, and LMxysn_1098, respectively. The open-reading frame (orf) LMxysn_0462 encodes for the InlA, which is a major factor required for internalization of Lm into the Caco-2 BBe cell line^16^.

The remaining two mutants harbored independent insertions within two closely placed CDS, LMxysn_1095, and LMxysn_1098. Bioinformatic analysis revealed that these genes are a part of an operon involved in the glycosylation of wall teichoic acid (WTAs), the main somatic antigen of Gram-positive bacteria. As both mutants display similar loss in invasive properties, we focused our attention on the gene LMxysn_1095, predicted to encode for an enzyme required for the transfer of unidentified sugar to the WTA.

**Fig. 4:**
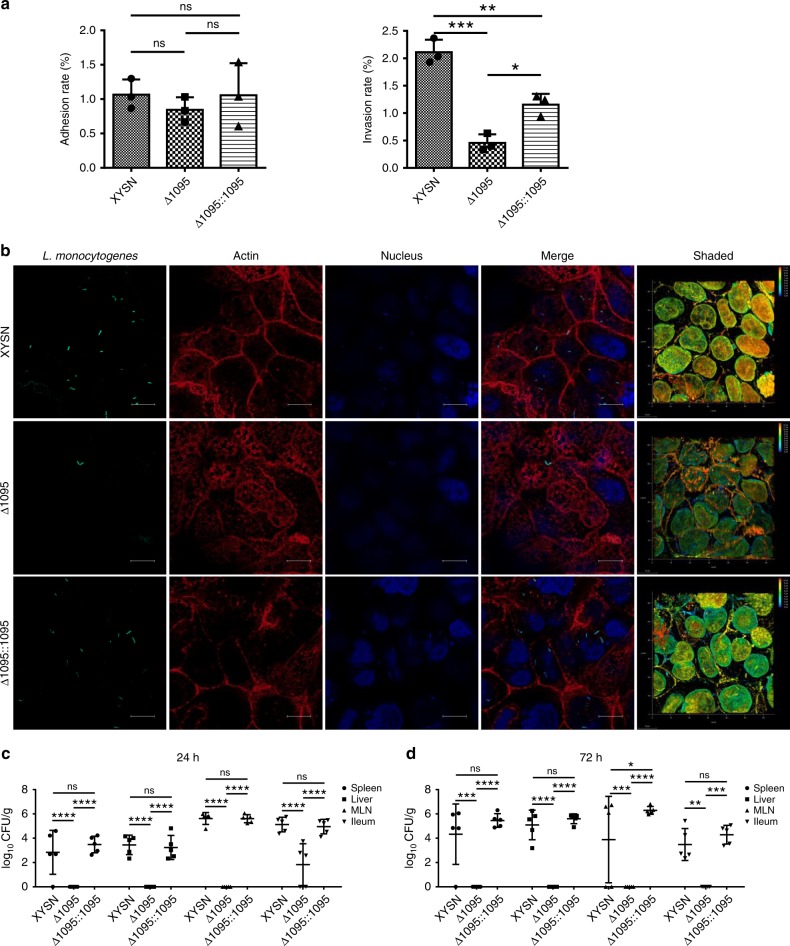
Comparative invasive capacities of XYSN and its isogenic WTA mutants. **a** Adhesion and invasion rates, and (**b**) confocal images of GFP-expressing strains XYSN, Δ1095, Δ1095::1095 in Caco-2 BBe cells at 15 min post internalization. The actin cytoskeleton was stained by phalloidin (red) and DNA was stained by DAPI (blue) following fixation and permeabilization of the sample. The fluorescent samples were analyzed by confocal microscopy. Magnification of all images: ×1000. Scale bars, 10 μm. Error bars represent SD, *n* = 3 independent experiments. Statistical analyses were carried out by Tukey's multiple comparisons test: *P < 0.05, **P < 0.01, ***P < 0.001, ns: no significance. **c**, **d** Comparative organ colonization properties of XYSN and its isogenic WTA mutants (Δ1095 and Δ1095::1095). Experiments were performed at 24 and 72 h post infection. Each dot represents an organ from one infected mouse. The log CFUs/g represents the mean of five mice per group. Statistical analyses were carried out by Tukey's multiple comparisons test: *P < 0.05, **P < 0.01, ***P < 0.001, ****P < 0.0001, ns: no significance. Error bars represent SD, data represent (**c**) and (**d**) two independent experiments

We generated an isogenic deletion LMxysn_∆l095 and examined its ability to adhere and invade Caco-2 BBe. Despite having similar adhesion levels as the parental strain (Fig. 4a; Supplementary Fig. 4, Supplementary Videos 1–3), there was a significant reduction in the number of LMxysn_∆l095 mutant bacteria internalized (Fig. 4a, b; Supplementary Videos 4–6). Complementation of the deletion mutant restored its invasive phenotype. We next examined the contribution of LMxysn_1095 to the virulence potential of XYSN in vivo using the mouse oral infection model. The ability of the mutant to colonize deeper tissues and internal organs was already severely compromised at 24 h post infection (p.i.); at 72 h p.i., no mutant bacteria could be recovered from the spleen and liver of the infected mice (Fig. 4c, d). In contrast, the parental XYSN colonized these organs to high levels even at 24 h p.i. Complementation of the mutant restored both entry and the virulence phenotype to that of the parental XYSN isolate. These results confirmed that LMxysn_1095 plays a key role in intestinal barrier translocation and successful organ colonization.

### Galactose decoration on WTA from the hybrid sub-lineage II isolate XYSN

**Fig. 5:**
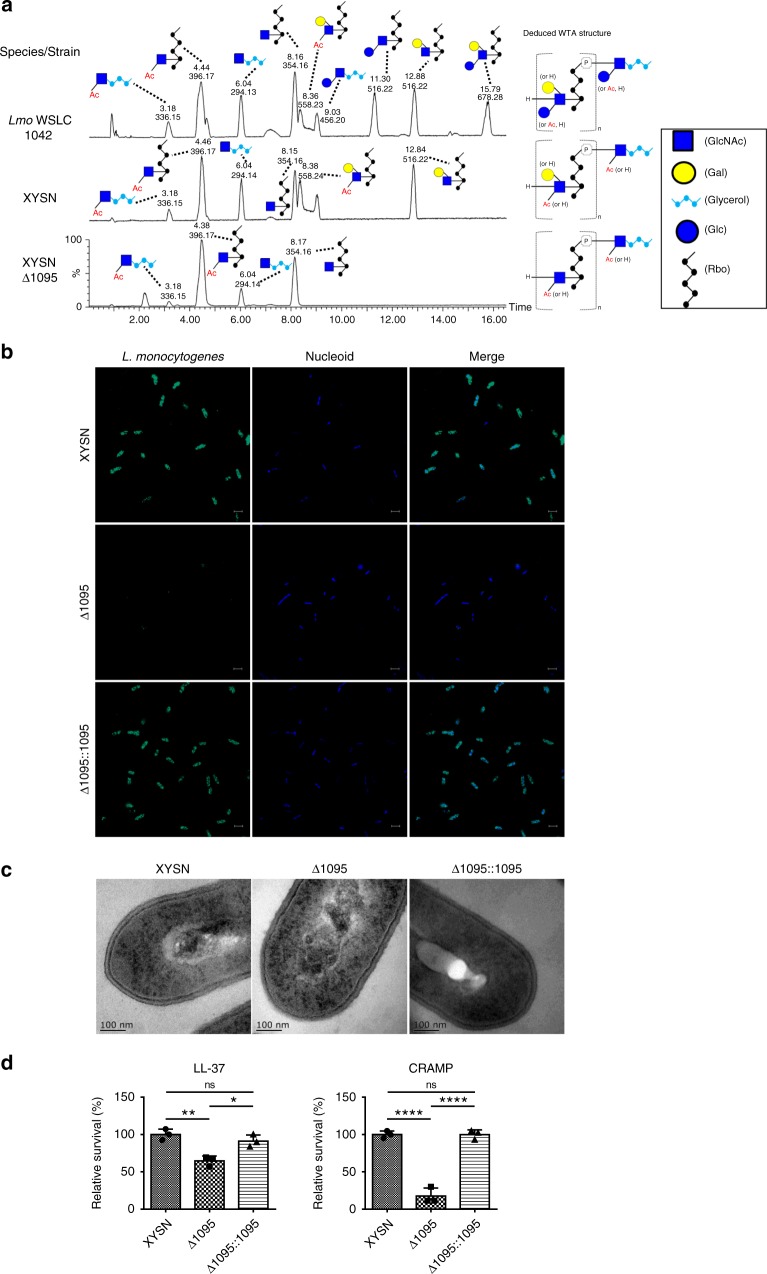
Molecular and structural features of wall teichoic acid of *L*. *monocytogenes* HSL-II strain XYSN. **a** Structure and UPLC-MS/MS analysis of type II WTA from *L*. *monocytogenes* WSLC 1042 serotype 4b strain as compared with XYSN and its isogenic Δ1095 mutant. Liquid chromatographic separation and MS-based identification of carbohydrate residues within *Listeria* WTAs from XYSN, Δ1095, and WSLC 1042. Peaks are labeled with their respective retention time (Rt;[min]) and base peak ion [M-H]^−^ (m/z). **b** Confocal images of XYSN, Δ1095, and Δ1095::1095. Bacteria were subsequently stained by *Listeria* O-antiserum 4 (primary antibody, 1:200) and Alexa Fluor 488-conjugated rabbit antibody (green), nucleoid was stained by DAPI (blue). Magnification of all images: ×3000. Scale bars, 2 μm. **c** Transmission electron microscopic images depicting intact cell wall structures of XYSN, rough cell wall structure of Δ1095 and regained intact cell wall of Δ1095::1095. **d** Role of galactosylated WTA in protection of bacteria towards antimicrobial peptides. Quantification of viable bacteria following treatment (2 h 37 °C) with LL-37 and CRAMP antimicrobial peptide (25 µg/mL each) on growing cultures. Statistical analyses were carried out by Tukey's multiple comparisons test: *P < 0.05, **P < 0.01, ****P < 0.0001, ns: no significance. Error bars represent SD, *n* = 3 independent experiments

Since WTAs are major antigenic determinants in defining serogroup specificity of *L*. *monocytogenes*, we examined the role of LMxysn_1095 in the agglutination reaction observed with serogroup 4-specific antiserum. The ∆l095 mutant did not agglutinate with this antiserum, a property that was restored when using the complemented strain, indicating that it is a major determinant of serogroup specificity (Supplementary Fig. 6). We note that even though XYSN and other HSL-II isolates are phylogenetically more closely related to lineage II strains, agglutination is obtained with an antiserum that classifies them as being related to lineage I serogroup 4 strains. To understand the basis of these observations, we extracted and purified WTA polymers from XYSN, LMxysn_∆l095, and the serotype 4b strain WSLC 1042, and determined their detailed structures using ultra-performance liquid chromatography-coupled electrospray ionization tandem mass spectroscopy (UPLC-MS/MS). The obtained chromatograms indicate that XYSN produces a type II WTA that is highly related to the carbohydrate species produced within the serogroup 4 strains. The glycosylation pattern of the GlcNAc in the 4b serovar strain involves both glucose and galactose, while only galactose is found to be associated with the GlcNAc moiety in the WTA of the XYSN (Fig. 5a). Deletion of LMxysn_1095 abrogates galactose decoration on WTA, indicating that it encodes a galactosyltransferase.

Confocal images of *L*. *monocytogenes* stained by fluorescein-labeled *Listeria* O-antiserum 4 indicated galactose decoration on WTA was associated with integrity of O-antigen (Fig. 5b). Transmission electron microscopy (TEM) of XYSN and the ∆l095 revealed ultrastructural changes in the surface of the cell wall of the mutant strain (Fig. 5c). The mutant was also more sensitive to the antimicrobial peptides (AMPs) LL-37 and CRAMP than the parental isolate. Resistance to AMPs was restored in the complemented mutant (Fig. 5d). Thus, the unique WTA structure plays a crucial role in XYSN’s resistance toward AMPs.

### Galactose glycosylation of WTAs promotes surface association of ActA, Ami, and InlA

**Fig. 6:**
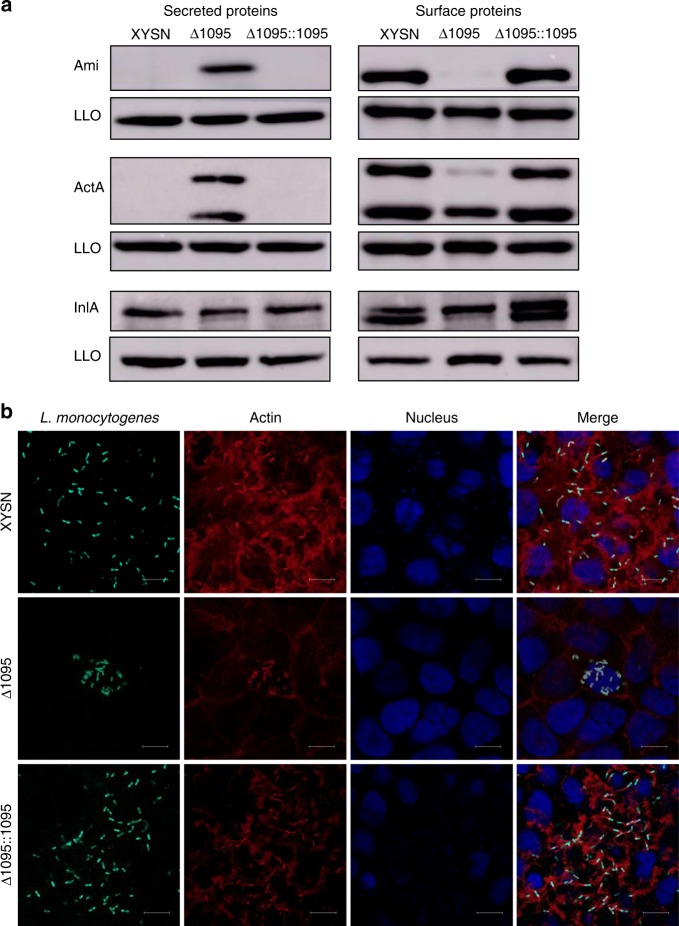
WTA galactosylation promotes association of Ami, ActA and InlA to the surface of Lm. **a** Surface-associated and secreted Lm proteins extracts were obtained from XYSN, ∆1095, and ∆1095::1095. LLO protein levels were used as sample loading control. The image presented is representative of three independent experiments. **b** Confocal images of XYSN, Δ1095, Δ1095::1095 in Caco-2 BBe cells at 7 h-post infection. ActA was visualized with an anti-ActA monoclonal antibody and goat Anti-Mouse Alexa Fluor 488-conjugated IgG (green), the actin cytoskeleton was stained by phalloidin (red), and DNA was stained by DAPI (blue) following fixation and permeabilization of the sample. Magnification of all images: ×1000. Scale bars, 10 μm

As WTA modification affects protein localization of surface proteins^17^, we examined the influence of galactose-based glycosylation on the location of specific virulence factors to the bacterial surface. Deletion of LMxysn_1095 showed redistribution of the GW-motif harboring proteins Ami and ActA from the cell surface to the secreted compartment (Fig. 6a). This was associated with a loss of “actin tail” formation and a defect in intracytoplasmic motility during infection of Caco-2 BBe cells (Fig. 6b). Complementation of the mutant restored association of ActA to the bacterial surface to levels seen in the parental isolate. The amount of InlA, a protein anchored to the cell wall by a LPXTG-containing motif domain, was also affected by the deletion of the galactosyltransferase encoded by LMxysn_1095 (Fig. 6a). Taken together, these data indicated that the galactose-based decoration of WTA is required for stable localization of important well-characterized virulence genes on the surface of the bacteria.

### *smcL* is required for the bi-zonal hemolysis of XYSN

**Fig. 7:**
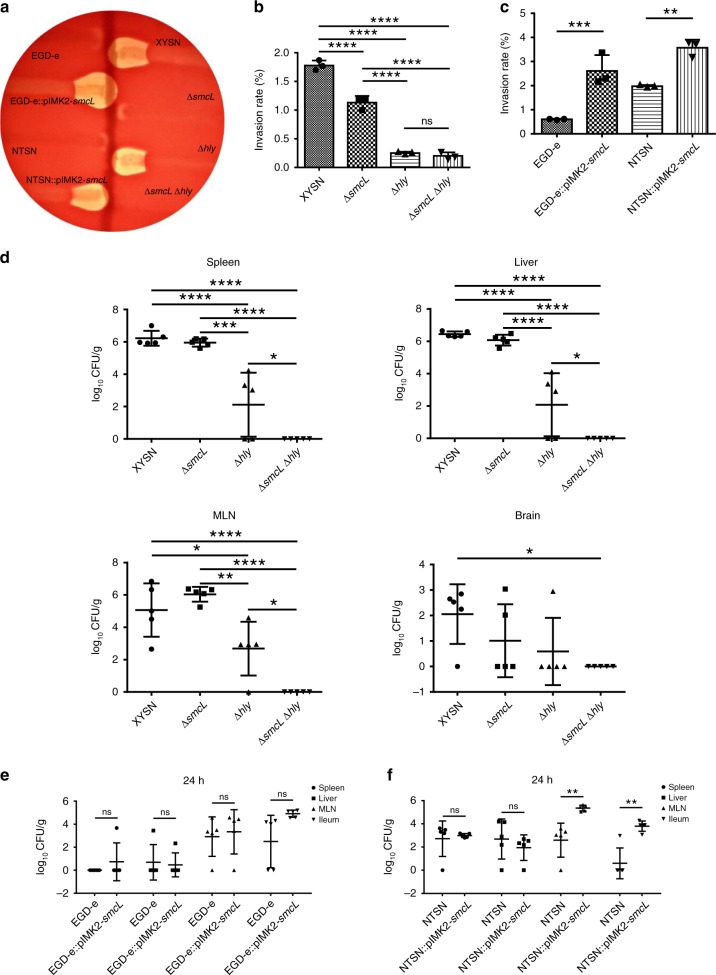
Contribution of *smcL* to the CAMP assay, invasion, and virulence. **a** The CAMP reaction on 5% sheep blood agar as observed after 36 h for XYSN, EGD-e, NTSN, Δ*smcL*, and Δ*hly* derivatives. *L*. *monocytogenes* EGD-e and NTSN exhibit typical zones of hemolysis, while XYSN also exhibits CAMP reaction indicating sphingomyelinase activity. XYSN lacking *smcL* exhibits only hemolytic zones, EGD-e::pIMK2-smcL and NTSN::pIMK2-smcL exhibit both hemolysis and sphingomyelinase activities. **b** Invasion rates of *L*. *monocytogenes* XYSN, Δ*smcL*, Δ*hly*, and Δ*smcL*Δ*hly* strains in the Caco-2 BBe cell model. Deletion mutants Δ*smcL* and Δ*hly* exhibited reduced invasion capability as compared with wild-type XYSN. Statistical analyses were carried out by Tukey's multiple comparisons test: ****P < 0.0001, ns: no significance. **c** Invasion rates of *L*. *monocytogenes* NTSN and EGD-e compared with their recombinant variants expressing *smcL*. The presence of the *smcL* promotes the invasive ability of the recombinant strains. Statistical analyses were carried out by Tukey's multiple comparisons test: **P < 0.01, ***P < 0.001. **d** Mice infection assay with XYSN and its isogenic mutants Δ*smcL*, Δ*hly*, and Δ*smcL*Δ*hly*. Experiments were performed at 72 h post infections. The log CFUs/g represents the mean of five mice per group. Each dot represents an organ from one infected mouse. Statistical analyses were carried out by Tukey's multiple comparisons test: *P < 0.05, **P < 0.01, ***P < 0.001, ****P < 0.0001. **e** Mice infection assay with EGD-e and its recombinant strain harboring *smcL*. The log CFUs/g represents the mean of five mice per group. Statistical analyses were carried out by Sidak's multiple comparisons test: ns: no significance. **f** NTSN and EGD-e recombinant strains harboring *smcL*. The presence of *smcL* improves translocation of the bacteria to the mesenteric lymph nodes and ileum. The *smcL* complemented lineage I strain NTSN exhibits superior activity as compared with the EGD-e complemented strain. The log CFUs/g represents the mean of five mice per group. Each dot represents an organ from one infected mouse. Statistical analyses were carried out by Sidak's multiple comparisons test: **P < 0.01, ns: no significance. Error bars represent SD, *n* = 3 independent experiments

The genes encoding listeriolysin (*hly*) and sphingomyelinase (*smcL*), two proteins with cytolytic properties, are located on two distinct pathogenicity islands, viz. LIPI-1 and -2 in XYSN, respectively. As the presence of the *sm*c*L* in the species Lm has not been studied, we first examined the relative contributions of the *hly* and *smcL* genes to the hemolytic and CAMP phenotype seen on blood agar plates. Both XYSN and its isogenic ∆*hly* mutant produced the typical shovel-shaped cooperative lytic reaction at the intersection with *R*. *equi* (Fig. 7a). Deletion of the *smcL* gene abrogated this property, and a double-deletion mutant XYSNΔ*smcL*Δ*hly* was both nonhemolytic and CAMP-negative when tested on blood agar plates. In order to have the comparative data, we determined the hemolytic titers of strains XYSN, EGD-e, and NTSN. The hemolytic titers of XYSN, EGD-e, and NTSN were similar (Supplementary Fig. 6). We generated recombinant strains EGD-e∷*smcL* and NTSN∷*smcL*, each harboring a copy of the *smcL* gene on the chromosome. Both strains exhibited shovel-shaped CAMP-like reaction with *R*. *equi*. Thus, the *smcL* gene of *L*. *monocytogenes* XYSN contributes to the strong bi-zonal hemolysis seen, an effect previously only observed for *L*. *ivanovii*.

### Role of listeriolysin and sphingomyelinase in the virulence of XYSN

As XYSN exhibits a strong invasive phenotype, we used the epithelial cell line Caco-2 BBe to assess the relative contributions of *hly* and *smcL* to invasion. Loss of the *hly* gene reduces the invasive ability of XYSN sevenfold and underlines a role of listeriolysin in promoting bacterial entry. For the mutant lacking the *smcL* gene, a decrease of ~1.6-fold in the invasion ability of the bacterium was observed (Fig. 7b). Introduction of *smcL* gene into EGD-e and NTSN increased the ability of these bacteria to invade the epithelial cell by at least twofold (Fig. 7c). Hence both *hly* and *smcL* promote entry of XYSN in human-derived intestinal epithelial cells.

We further examined the respective roles of *hly* and *smcL* in the mouse infection model. For the ∆*smcL* mutant, there was no significant reduction of bacteria present in either spleen or liver. In contrast, the ∆*hly* mutant was strongly impaired in the ability to colonize these organs (Fig. 7d). The double-mutant lacking both the *hly* and *smcL* genes was even further restricted in its ability to colonize both the spleen and liver (Fig. 7d).

We also assessed the contribution of *smcL* on bacterial translocation at an early time point following oral infection using recombinant strains. Introduction of *smcL* into the serotype 1/2a EGD-e strain promoted colonization albeit to a lesser extent than that seen with the NTSN strain (Fig. 7e). Introduction of *smcL* in the serotype 4b NTSN strain significantly increased the invasive and colonization abilities of this bacteria and their migration to the mesenteric lymph nodes at 24 h p.i. (Fig. 7f). Taken together, these results demonstrate that *smcL* has a role in both invasion and virulence of XYSN.

## Discussion

*L*. *monocytogenes* is one of the deadliest foodborne pathogens known and there is presently concern that foodborne outbreaks are increasingly caused by hypervirulent isolates^9,18^. Genome-based population analysis of a large number of historical isolates from diverse sources and geographical locations collected over the past 90 years, define four clearly separated major lineages within this species. Surprisingly, not one of the 15 species discovered over the past 20 years, classify as pathogens emphasizing the unique virulence potential of the species Lm. The isolates described in this study are unique in several ways: (i) they are unable to ferment rhamnose that is one of the four identifying biochemical features of Lm, (ii) they exhibit a CAMP-positive phenotype, typically only seen with the species *L*. *ivanovii*, (iii) they possess distinct anionic surface structures defining the serotype 4h, (iv) they are the only known Lm to carry genomic segments characteristic of another species, viz., *L*. *ivanovii*, and (v) they are the most virulent representatives of this species known today. None of the Lm isolates described to date has any of the properties described above nor do they exhibit the genetic diversity seen with the isolates described here. Taking into the account that these isolates have acquired properties from different lineages and species, and given their phylogenomic isolation in a unified but distinct clade adjacent to lineage II, we have designated these isolates as members of the hybrid sub-lineage of the major lineage II.

Regulation of the expression of virulence genes during infection is critical for the outcome of disease and is therefore strongly controlled. Expression of virulence genes including products of the LIPI-1-and the *inl*AB- loci are controlled by the transcriptional regulator PrfA^19,20^. Previous studies have established that a Gly145Ser (PrfA*) mutation in the PrfA regulator leads to the constitutive expression of several virulence genes^21,22^. The Lm_XYSN PrfA does not carry this mutation or other commonly occurring mutations (K156E, T165A, K197N, and C229Y), and it is unlikely that an increased expression of LIPI-1 gene products and/or InlAB underly the hypervirulent phenotype observed^23^. Indeed, the hemolytic titer of XYSN is similar to that of the Lineage II strain EGD-e, and slightly lower than that of the Lineage I strain NTSN (Supplementary Fig. 6).

Previous studies linking species and lineage heterogeneity of Lm virulence factors to severity of clinical disease have revealed the presence of strongly structured major sublineages in lineages I and II, and led to the detection of a hypervirulent clone in Lineage I harboring a novel pathogenicity island (LIPI-4)^9^. However, HSL-II isolates do not harbor the LIPI-4 locus. Another pathogenicity island, LIPI-3 which is present in ~60% of all lineage I strains, is also absent in HSL-II isolates^9^. Other more distant sublineages in Lineage II, designated SL842 and SL843, share only three of the 31 RODs with HSL-II isolates^8^. As detailed here, the virulence attributes of HSL-II isolates are due to a unique composition of wall teichoic acids whose genetic origins are in lineage I, and the *smcL* gene LIPI-2 locus acquired from another pathogenic species *L*. *ivanovii*. Our studies indicate that it is the synergistic interactions between these factors and other PrfA-regulated core virulence factors encoded by the LIPI-1 locus and InlAB locus that underly the hypervirulence seen with HSL-II isolates.

HSL-II isolates possess a number of traits that are unusual for the species Lm: they are rhamnose-negative, exhibit a CAMP phenotype, are agglutinated only by serogroup 4 antisera, and are untypable by a widely used PCR-based serogrouping scheme. The operon required for utilization of rhamnose is absent in XYSN, 15LG, and 16E strains, and the genome organization at this chromosomal region resembles that seen for other rhamnose-negative *Listeria* species e.g., *L*. *seeligeri* (Supplementary Fig. 7). The CAMP phenotype is due to the enzymatic activity of the sphingomyelinase present on the acquired LIPI-2 element from *L*. *ivanovii*. Finally, of the five genes *prs*, *lmo737*, *lmo1118*, *orf2110*, and *orf*2819 used in the in silico serogrouping scheme of Doumith et al.^7^, XYSN lacks three loci, viz., *lmo*737, *lmo1118*, and *orf*2819 (Supplementary Fig. 8).

Comparative genome analysis indicates that the XYSN-WTA cluster is derived from lineage I serotype 4b strains (Supplementary Figs. 9, 10). In contrast, genes associated with WTA production in lineage II serotype 1/2a strains are similar to those present in the species *L*. *seeligeri* (Supplementary Fig. 11). The WTA glycopolymer of XYSN is encoded by a unique cluster of genes (LMxysn_1090–1104), that is absent in EGD-e, a lineage II strain. Elucidation of its detailed molecular structure reveals that it resembles the type II WTA structure of a serogroup 4 and involves decoration of GlcNAc with galactose. Deletion of the galactosyltransferase encoded by LMxysn_1095 of the WTA operon causes loss of agglutination with serotype 4 antisera indicating an essential role of this modification for antigenicity. These data explain why the antigenic profile of HSL-II isolates resemble that of lineage I isolates.

Wall teichoic acids (WTAs) in the surface of Gram-positive bacteria play a crucial role in the maintenance of the integrity of the bacterial envelope, in host defense evasion, and mediating virulence^24,25^. For the lineage II EGD-e strain, loss of decoration of its WTA with L-rhamnose results in re-localization of the surface-bound virulence factor Ami, which harbors domains encoding the cell-wall associating GW-motif^17^. Here, we extend these observations and show that the spatial distribution of ActA, which is anchored to the cell membrane by a transmembrane domain unrelated to the GW-motif, is strongly altered in the ∆1095 mutant strain. Thus, galactosyl-dependent decoration of WTA has important consequences for listerial physiology ranging from induction of physiological levels of autolysis, to loss of ActA-mediated intracellular motility, and can account for the attenuated virulence of the Δ1095 mutant strain.

Infection studies with mice indicate an essential role for galactosylated WTA in virulence. These results are in keeping with previous reports on the role of WTA decorations in the pathogenesis of various lineages of Lm^26–29^, and underline the maintenance of a specific WTA structure in virulence. Studies on a highly oral-virulent strain F6214-1 revealed an essential role of galactosylated WTA in orally infected mice^29^. It has been previously shown that WTAs protect bacteria against the activities of antimicrobial peptides (AMPs)^30^. Here, we show that the galactosylated WTA moiety is critical in protecting the bacteria against AMPs and requires the galactosyltransferase activity encoded by LMxysn_1095. Thus, interruption of WTA decoration by targeting this protein could be an effective strategy to limit infections caused by HSL-II isolates.

A role for the *hly* gene encoding the pore-forming toxin listeriolysin in cellular invasion has been previously reported^31,32^. For XYSN, we demonstrate that both LLO and SmcL promote entry into the epithelial cells. Significantly, introduction of the *smcL* gene into lineage I (NTSN) and lineage II (EGD-e) strains not only enhanced the invasive properties for Caco-2 BBe cells but also improved the ability of these recombinant strains to translocate to deeper tissues in the gut following orogastric inoculation. Our studies with the guinea pig model suggest that additional synergistic mechanisms that significantly enhance InlA-dependent translocation from the alimentary tract exist, and indicate that HSL-II isolates reported here are useful model organisms to uncover these additional pathways and to discern their contribution to the severe disease in the different tissular compartments.

**Fig. 8:**
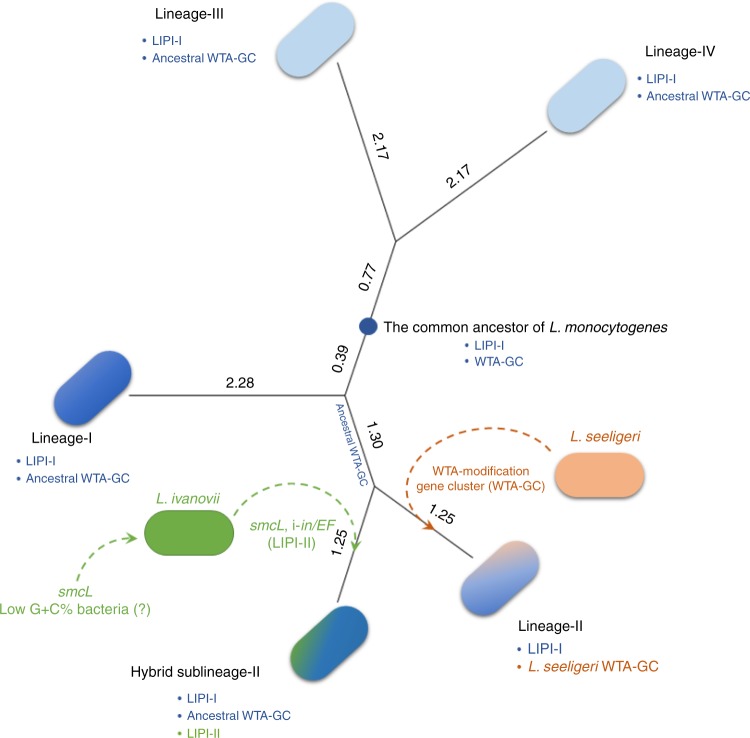
Evolutionary model of *L*. *monocytogenes* HSL-II. The phylogram is constructed using MUMmer-based average nucleotide identity (ANIm) of closed genomes of all lineages. The lineages are proposed to originate from a common ancestor possessing a 4b-like wall teichoic acid gene cluster (WTA-GC). For Lineage II, subsequent recombination events led to replacement of WTA-GC from *L*. *seeligeri*. Lineage II and HSL-II then separated and followed independent evolutionary paths. Ecological coexistence of HSL-II with other Lm lineages and *L*. *ivanovii* led to exchange and acquisition of genetic clusters, including a partial LIPI-II locus. The number next to each clade represents the %ANIm difference from respective last common ancestor. The topology of the tree is identical to the phylogenomic tree calculated based on the core genome (as in Fig. 2). WTA-GC: wall teichoic acid gene cluster

In conclusion, this report document the presence of Liv-associated virulence genes in Lm and suggest that precursor species of the genus *Listeria* as well as other related bacteria co-existed in common environments (Fig. 8). Significantly, HSL-II isolates share many core virulence factors found in isolates of the other highly virulent Lm lineages I and II, implying an association with human cases of listeriosis. Its unusual properties, particularly its rhamnose-negative phenotype, would have precluded their recognition as bona fide members of the species Lm and may resulted in them being overlooked. Finally, our study suggests that hitherto undiscovered variants of Lm in addition to those in the pathogenic lineages I and II probably exist and warrant further research to identify and detect additional lineages of hypervirulent Lm.

## Methods

### Bacterial strains, cell lines, and animals

Wild-type strains and plasmids are listed in Supplementary Table 1. *L*. *monocytogenes* strains NTSN, XYSN, and 15LG were isolated from three different outbreaks in 2011, 2012, and 2015, respectively, from either brain tissue or cerebrospinal fluids of goats diagnosed with meningitis. Isolate 16E was isolated from the environment of a goat-rearing farm in 2016. The isolates of XYSN, 15LG, and 16E were collected from different farms that are separated by about 50 km. All the mutant strains and recombinant strains used in this study are listed in Supplementary Table 2. Primers used to amplify genes are listed in Supplementary Table 3. The human epithelial colorectal adenocarcinoma cell line ATCC CRL-2102 Caco-2 BBe was propagated in the Dulbecco's modified Eagle medium (DMEM, Gibco) supplemented with 10% fetal bovine serum (FBS, CLARK). Six-week-old female C57BL/6, BALB/c mice, and female guinea pigs (150 g) were purchased from Vital River Laboratory Animal Technology Co., Ltd. (Beijing, China). Animal experiments were conducted in accordance with guidelines laid down for the welfare and ethics of experimental animals. All animals were kept at the animal biosafety facilities according to procedures approved by the Institutional Animal Ethics Committee of Yangzhou University.

### Phenotypic characterization

Samples from brain tissue and cerebrospinal fluid were inoculated onto CHROMagar^TM^ plates. Following overnight cultivation, blue/green colonies with opaque halos were confirmed as *L*. *monocytogenes* by biochemical assays performed with VITEK^®^ 2 GN card (Biomerieux, France) for identification. PCR-based serotyping was carried out by multiplex PCR method^7^ and slide agglutination using *Listeria* O and H antisera (Denka-Seiken Co. Ltd., Tokyo, Japan; Becton, Dickinson and Company, Sparks, MD, USA) following instruction of the vendors.

Antimicrobial susceptibility testing was performed against 13 different antimicrobials using a disc-diffusion method as recommended by the Clinical and Laboratory Standards Institute^33^.

The invasion potential of *Listeria* strains XYSN, 15LG, 16E, EGD-e, LM11-00412, LM13-00344, LM6-01023, NTSN, and *L*. *ivanovii* ZJU was examined using the human colon cancer epithelial InlA-dependent cell line Caco-2 BBe [MOI (ratio of bacteria to cells) = 20]. Cultures were grown by inoculating a single colony in BHI broth following overnight incubation at 37 °C. Cultures were transferred to fresh 10-ml BHI broth (1:50 ratio) and grown at 37 °C until reach an OD_600_ value of 0.8. Bacterial cells were washed twice and appropriately diluted to 1 × 10^7^ CFU/mL in the DMEM medium. Caco-2 BBe were trypsinized, seeded in 24-well plates, and cultured for 12–16 h to form monolayers with ~90% confluence. Cell monolayers (5 × 10^5^ cells) were infected with bacteria at a MOI of 20 for 1 h, after which the medium was removed, and the cells washed twice with PBS. This was followed by the addition of 1 mL of DMEM medium containing 50 μg/mL gentamicin, and incubation for a further 15 min to assess invasion ability^16^. Cells were subsequently washed twice with PBS, and 0.2% Triton X-100 was added and incubated for 8–10 min at room temperature for cell lysis. Lysates were serially diluted with PBS and plated onto agar plates and the bacterial numbers determined following incubation for 20 h at 37 °C, and the bacterial invasion rate was calculated with the following formula: (number of bacteria internalized/number of initial bacteria in wells) × 100%.

Kaplan–Meier plots of 6-week-old female C57BL/6 mice (*n* = 6/group) were used to estimate survival rates following intra-gastric (i.g.) inoculation with 2 × 10^8^ CFU^34^. Organ colonization properties of XYSN, EGD-e, LM11-00412, LM13-00344, LM6-01023, and *L*. *ivanovii* ZJU (*n* = 7/group) were evaluated at 48 h post infection (p.i.) by determining bacterial loads in the MLN, ileum, spleen, and liver of mice. Ileum samples were treated as follows: a 2-centimeter-length ileum was taken and washed by a syringe with 5 mL of PBS to remove the luminal contents. The ileum was homogenized, and serial dilutions of the organ homogenates plated onto CHROMagar plate. The lower limit of detection for these CFU assays was 100 CFU/gram. In addition, the weight changes and bacterial loads in organs as well as brain were determined at 120 h p.i. The respective inoculation doses used for evaluating organ colonization are: XYSN and 16E (2 × 10^6^ CFU), 15LG (1 × 10^6^ CFU), 2 × 10^8^ CFU for EGD-e, NTSN, LM11-00412, LM13-00344, LM6-01023, and *L*. *ivanovii* ZJU, respectively. In total, 150 g-weight female guinea pigs (*n* = 10/group) were orogastrically inoculated with XYSN and EGD-e. The inoculation doses were 1 × 10^9^ CFU bacteria resuspended in 3 mL of CaCO_3_ solution (30 mg/mL). The survival of guinea pigs was observed and recorded continuously for 14 days. The experiments were repeated twice.

### Transposon mutagenesis

The *Mariner* family transposable element TnYLB-1 was introduced into competent XYSN by electroporation. Transposon-marked mutants were identified by their resistance to kanamycin and sensitivity toward spectinomycin. From the XYSN transposon mutant library of ~12,000 individual mutants, 1127 mutants were randomly selected for further study. The mutant-screening procedure was established using Caco-2 BBe, where the invasive capacity of wild-type and mutant bacteria was compared using a MOI of 20. Mutants with a fivefold lower invasion rate were further analyzed to identify location of the insertion. Briefly, DNA from mutant strains were digested by restriction enzyme *Taq*I and circularized by DNA ligation. The target fragments were amplified using an inverse PCR protocol with primers oIPCR1 and oIPCR2, and the point of insertion in the genome identified following sequencing by alignment to the genome^35^.

### Mutant strains construction and complementation studies

The upstream and downstream homology fragments of the LMxysn_1095 gene (henceforth referred as 1095) were amplified, purified, and ligated to the thermo-sensitive replacement vector pAULA for gene replacement^36,37^. Following identification of the desired clone, the recombinant pAULA plasmid was electroporated to XYSN to generate the mutant strain Δ1095. The complemented strain Δ1095::1095 was obtained by replacement of a 1225 bp-tagged fragment of the parental strain of Δ1095. Replacement of the tag with CACATT, instead of CATATA, following the termination codon was used to confirm the reversion (Supplementary Table 3). Strains expressing GFP were generated by electrotransformation plasmid pERL-3-cspl-GFP.

The mutant strains XYSNΔ*smcL*, XYSNΔ*hly* were obtained using the strategy described above for the mutant strain Δ1095. The XYSNΔ*smcL*Δ*hly* mutant was obtained by deleting the *smcL* from XYSNΔ*hly*. The positive clones were further verified by sequencing. EGD-e::pIMK2-*smcL* and NTSN::pIMK2-*smcL* recombinants were generated using the *hly* promoter and its signal peptide sequence to drive expression of the *smcL* gene.

### Surface and secreted proteins of ΔLMxysn_1095

Lm protein extracts were analyzed by SDS-PAGE and immunoblotting^19^. Supernatant and cell wall proteins were extracted from bacterial cultures growing at OD_600_ = 0.8. Protein concentrations were determined using the BCA Protein Assay Kit (Beyotime) and used at 30 µg/well. Proteins were detected using the following antibodies: mouse monoclonal antibody against LLO (3B6) and ActA (6F5) at a 1:2000 dilution, and rabbit polyclonal anti-InlA serum (CSB-PA758331HA01AAD, CUSABIO) at 1:2000 dilution and anti-Ami serum at a 1:5000 dilution^38–40^. HRP-labeled goat anti-mouse IgG and goat anti-rabbit IgG were used as secondary antibodies.

The resistance activity of XYSN, Δ1095, and Δ1095::1095 to antimicrobial peptides (AMPs) was determined in vitro^24^. Bacteria were harvested at a density of 0.5–1.0 × 10^9^ CFU/mL (OD_600_ = 0.8), and diluted with PB buffer (10 mM phosphate buffer, 1% BHI, pH 7.4). Equal volumes of 5 × 10^6^ CFU/mL bacteria and 25 µg/mL antimicrobial peptides CRAMP or LL-37 (GL Biochem) were then mixed in a 96-well microplate and incubated for 2 h at 37 °C. Mixtures were serially diluted and plated on to BHI agar plates with overnight incubation at 37 °C to determine bacterial counts. These experiments were repeated thrice.

The ultrastructure of wild-type, mutant, and complemented strains was observed using transmission electron microscopy. Exponential-phase bacteria were fixed with 2.5% glutaraldehyde overnight at 4 °C, dehydrated after incubation with graded concentrations of ethanol, and washed with 100% acetone. Samples were subsequently treated with acetone and resin, and finally embedded with pure resin for polymerization. Samples were ground and cut using an ultramicrotome, and successively stained with uranyl acetate for 25 min and lead citrate for 15 min. Images were acquired with a Tecnai 12 transmission electron microscope (Philips, New Zealand).

Extraction of cell wall components and purification of WTA was performed as described^41^. XYSN, ∆1095, and reference strain *L*. *monocytogenes* WSLC 1042 were cultured in BHI broth. Exponentially growing culture (OD_600_ = 0.8) was heat-killed by steaming for 30 min, harvested, and resuspended in SM buffer (100 mM NaCl, 8 mM MgSO_4_, 50 mM Tris, pH 7.5). Cells were disrupted by passing through a French press at 18,000 psi twice, the cell debris was removed by centrifuge, then the resuspended cell walls were treated with RNase and DNase, and subsequently treated with proteinase K. The cell wall preparations were harvested, freeze-dried, and stored at −70 °C. The crude WTAs were dialyzed through the Tube-O-DIALYZER^TM^, and purified anion exchange chromatography on a HiTrap DEAE FF column. The crude WTAs were extracted via incubation in a Falcon^TM^ tube, and further purified with anion exchange chromatography on a HiTrap DEAE FF column. Finally, purified WTA polymers were cleaved to monomeric units by 48% hydrogen fluoride (HF). The composition of WTA monomers was analyzed with ultra-performance liquid chromatography tandem mass spectrometry (UPLC-MS/MS).

To examine for adhesion and internalization, listerial strains GFP-carrying labeled with the plasmid pERL-3-cspl-GFP were fixed on the glass slides, permeabilized, and stained with buffer containing Phalloidin-iFluor 555 (Abcam, London, England). Slides were subsequently incubated at room temperature (RT) for 40 min prior to washing twice with PBS, followed by DAPI staining. To observe cytosolic motility, Caco-2 BBe cells were examined at 7 h post infection (MOI = 20:1) and labeled with ActA-specific antibody and goat anti-mouse IgG-conjugated with Alexa Fluor® 488 (Abcam, London, England). Three-dimensional reconstruction of images from multiple optical sections was accomplished using LAS X Software on a Leica-SP8ST-WS computer.

Orogastric virulence assays were performed with 6-week-old female BALB/c mice (*n* = 5/group) using a dose of 5 × 10^5^ CFU. Bacteria in the ileum, mesenteric lymph nodes (MLN), spleen, and liver were enumerated on day 1 and 3 post inoculation following tissue homogenization and serial plating. The animal experiments were conducted twice.

### The role of *smcL* in invasion and colonization

The supernatants of overnight grown cultures were collected by centrifugation, and OD_600_ adjusted using PBS buffer. Seventy microliters of each supernatant was added to a 96-well plate, followed by twofold serial dilution steps. Subsequently, 30 μL of 1% sheep erythrocytes were added, and hemolysis was observed after 1 h at 37 °C. The hemolytic titre was scored as the highest dilution required for 50% lysis of the erythrocyte suspension added^42^.

Hemolytic activity of XYSN, Δ*smcL*, Δ*hly*, Δ*smcL*Δ*hly*, EGD-e, EGD-e∷pIMK2-*smcL*, NTSN, and NTSN∷pIMK2-*smcL* were determined on 5% sheep blood agar. *R*. *equi* was streaked vertically, while *Listeria* strains streaked out perpendicularly on the plate^43^. Plates were incubated at 37 °C for 36 h and examined for zones of hemolysis.

The animal infection experiment was same as described in method 4.5, except using a dose of 2 × 10^6^ CFU. Bacteria in the mesenteric lymph nodes (MLN), spleen, liver, and brain were enumerated at day 3 post inoculation. EGD-e, EGD-e∷pIMK2-*smcL*, NTSN, and NTSN∷pIMK2-*smcL* were inoculated at a dose of 1 × 10^9^ CFU. Bacteria in the ileum, MLN, spleen, and liver were enumerated on day 1 post inoculation. The animal experiments were repeated twice.

### WGS and bioinformatic analysis

The closed genome of *L*. *monocytogenes* XYSN was obtained using 454 pyrosequencing-based technology. In brief, bacterial DNA was isolated from 18 h old cultures using the TIANamp Bacteria DNA kit following instructions of the manufacturer. DNA sequencing libraries were prepared using the GS FLX Titanium General Library Preparation kit, and paired-end sequencing with a read-length of ~450 bp was performed using the Roche 454 platform at the Chinese National Human Genome Center in Shanghai. A total of 148,335 reads were obtained and assembled by 454 software Newbler version 2.3 that resulted in 90 contigs (≥500 bp). Contigs were mapped against *L*. *monocytogenes* EGD-e and the gaps were closed using PCR-based mapping and sequencing. The closed genome was annotated by PROKKA v1.12 using a manually generated database of curated genomes of *L*. *monocytogenes*^44^.

For the isolates 15LG and 16E, DNA libraries were prepared using SPARK DNA Sample Prep Kit following the manufacturer’s instruction, and sequenced on HiSeq XTen sequencing system (Illumina, San Diego, USA). Reads were cropped using Trimmomatic v0.36^45^ by setting a sliding-window of 4:15, a minimum Phred score of 33, and removal of adaptor sequences and leading and trailing low-quality bases (below quality 3), as well as reads <36 bp. Subsequent read quality was determined by FastQC (http://www.bioinformatics.babraham.ac.uk/projects/fastqc) and de novo assembled using SPAdes v3.12.0^46^. The resulting contigs of >500 bp length and >5x average k-mer read coverage were mapped against the closed genome of XYSN using a MAUVE v2.4.0 tool^47^.

### Comparative genome analysis

For comparative analysis, we used publicly available (January 2018) closed genomes of 144 strains representing all the known four lineages (Supplementary Table 4). Average nucleotide identity (ANI) was calculated by pyANI using “blastall” (legacy BLAST) as well as “ANIm” (mummer) option^48^. The in silico DNA–DNA hybridization quotient was determined using genome-to-genome distance calculator (GGDC) formula-2^49^. Genome sequences were analyzed for CRISPR elements by a CRT v1.1 tool^37^, prophages by the PHASTER server^50^, and IS sequences by the ISfinder server^51^. The virulence genes were identified by blastp against latest “Virulence Factor Database” (VFDB)^52^ using 80% coverage and 70% protein identity cutoff. The genome was visualized, compared, and analyzed in Ugene v1.29^53^. To determine the multi-locus sequence type (MLST), sequences of respective genes were extracted from genome as well as PCR amplified and re-sequenced for the confirmation of altered nucleotides, if any. The resulting sequences were then assigned to MLST type using http://bigsdb.pasteur.fr/listeria/. The pan-core genome analysis was determined by Roary v3.12.0^54^ using the minimum percentage identity at 70%. The resulting core genome alignment from Roary was used to infer phylogeny using RAxML v8.0^55^ conducted with 100 bootstraps. Output trees were visualized by Figtree v1.4.3 (http://tree.bio.ed.ac.uk/software/figtree/). SNPs were determined using snippy v3.5.2 (https://github.com/tseemann/snippy).

Core genome MLST (cgMLST) was carried out using the chewBBACA suite v2.0.13^17^. In brief, a whole-genome locus sequence typing scheme was created (“CreateSchema” option in chewBBACA suite) using CDS from closed genomes of 144 isolates and the HSL-II isolate XYSN. Only loci present in least in 99% of the isolates were considered for the cgMLST-based scoring.

### Statistical analysis

Statistical analyses were performed with Prism 6 (GraphPad Software, version 8). One-way ANOVA was used with Tukey's multiple comparisons test for pairwise comparison of means from more than two groups, or Dunnett's multiple comparisons test for comparison of means relative to the mean of a control group; two-way ANOVA with Tukey's or Sidak's multiple comparisons test was used to compare the means of two groups. Statistically significant differences are: *P < 0.05; **P < 0.01; ***P < 0.001; ****P < 0.0001. Statistically nonsignificant (ns) was denoted when *p*-value was >0.05.

### Reporting summary

Further information on research design is available in the Nature Research Reporting Summary linked to this article.

## Supplementary information

Supplementary Information

Description of Additional Supplementary Files

Supplementary Movie 1

Supplementary Movie 2

Supplementary Movie 3

Supplementary Movie 4

Supplementary Movie 5

Supplementary Movie 6

Reporting Summary

Source Data

## Data Availability

The authors declare that all the data supporting the findings of this study are available within the article and its Supplementary Information files. The data underlying Figs. 1–8 as well as Supplementary Figs. 1, 3, 4, 5, and 6 are provided in a “Source Data” file. The sequencing data for *L*. *monocytogenes* XYSN is deposited and publicly available under the bioproject accession number PRJNA244955. The sequence reads for the isolates 15LG and 16E are deposited and publicly available under the bioproject accession number PRJEB27754. The accession number of xysn_1095 is MH939249. All other data are available from the corresponding authors upon reasonable requests.
