# Supplementary material for: A hybrid sub-lineage of Listeria monocytogenes comprising hypervirulent isolates
Source: Nat Commun. 2019 Sep 30;10:4283. doi: 10.1038/s41467-019-12072-1 (PMC6768887; doi:10.1038/s41467-019-12072-1)
Supplement: Supplementary file 1 — Supplementary Information [file 41467_2019_12072_MOESM1_ESM.pdf]

A hybrid sub-lineage of *Listeria monocytogenes* comprising hypervirulent isolates

Yin et al.

Supplementary Material

Supplementary Figure 1 : Comparative virulence of XYSN, 16E, 15LG, EGD-e, NTSN, LM11-00412, LM13-00344, LM6-01023 and *L. ivanovii* ZJU. (A, B) Mouse body weight change on day 3 and day 5 after infection; (C, D, E, F) Bacteria load in the organs at 5 days post-infection. Female C57BL/6 mice were orally inoculated, the dose of Lm: 2×10^6 CFU for XYSN and 16E; 1×10^6 CFU for 15LG (1 mice was dead during infection); 2×10^8 CFU for EGD-e, NTSN, LM11-00412, LM13-00344, LM6-01023 and *L. ivanovii* ZJU (n = 7). Each dot represents an organ from one infected mouse.

A

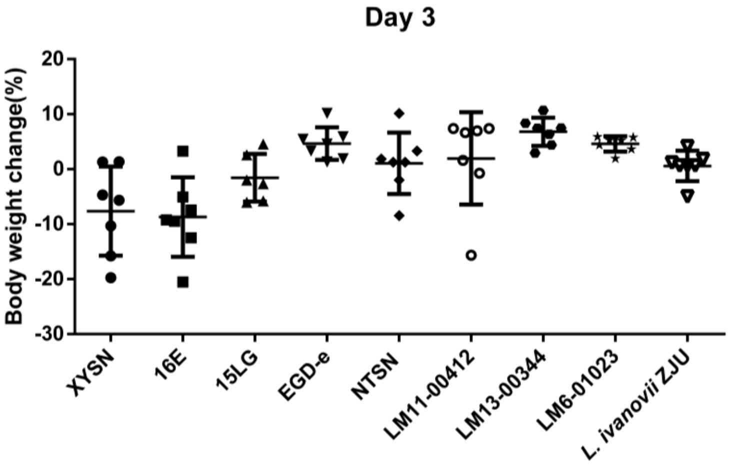

B

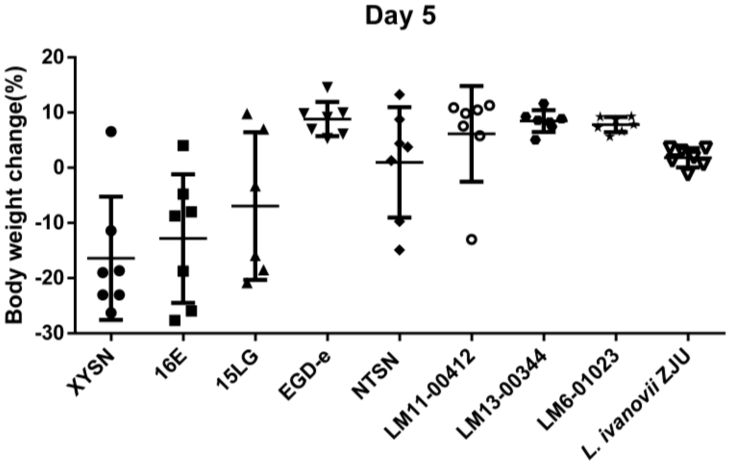

C

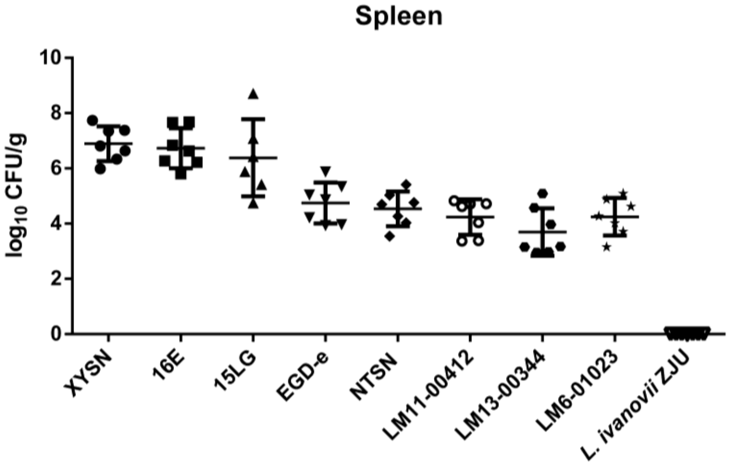

D

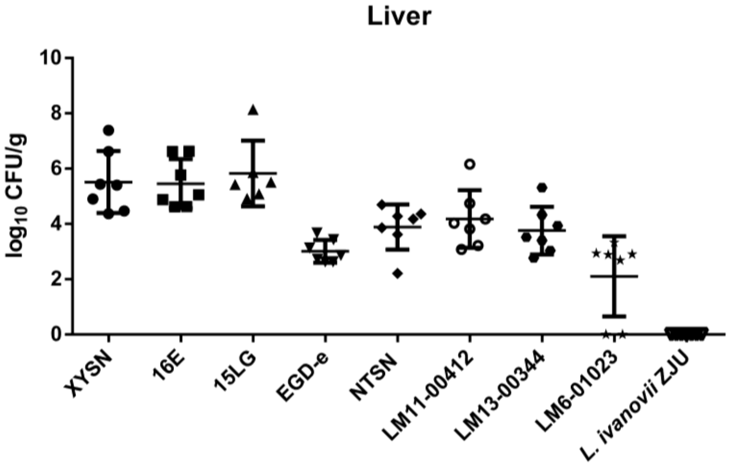
Liver
log10 CFU/g
10
8
6
4
2
0
XYSN
16E
15LG
EGD-e
NTSN
LM11-00412
LM13-00344
LM6-01023
L. ivanovii ZJU

E

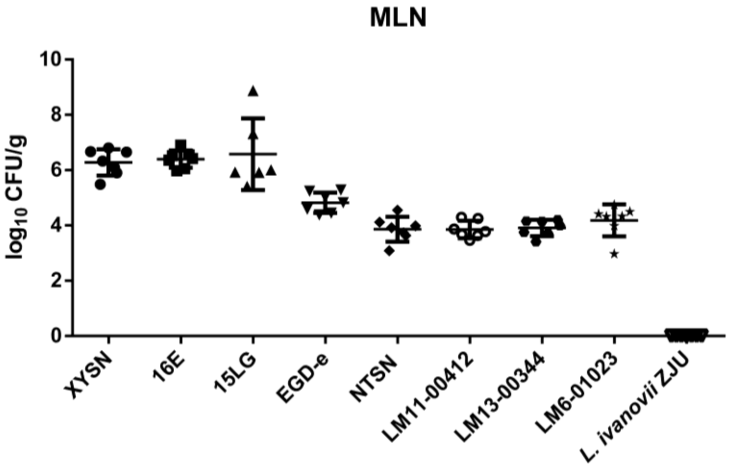
MLN
log10 CFU/g
10
8
6
4
2
0
XYSN
16E
15LG
EGD-e
NTSN
LM11-00412
LM13-00344
LM6-01023
L. ivanovii ZJU

F

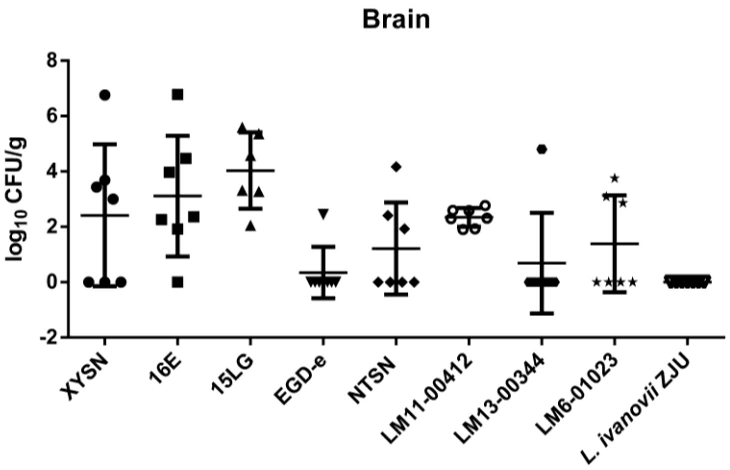
Brain
log10 CFU/g
8
6
4
2
0
-2
XYSN
16E
15LG
EGD-e
NTSN
LM11-00412
LM13-00344
LM6-01023
L. ivanovii ZJU

Supplementary Figure 2: Chromosomal region *L. monocytogenes* XYSN showing partial LIPI-II and its comparison to corresponding region in *L. ivanovii* WSLC3009. Three of the twelve genes (*smcL*, *i-inlF*, *i-inlE*) of LIPI-II locus defined in *L. ivanovii* were present in HSL- II isolates. The tRNA-Arg was located upstream while IS256 and IS3 family transposases were located downstream to this partial LIPI-II. The acetyltransferase gene present adjacent to both these transposases on the XYSN chromosome shows highest sequence similarity to *L. ivanovii*. The *L. ivanovii* WSLC3009 region was adapted from Dominguez-Bernal et al., 2006.

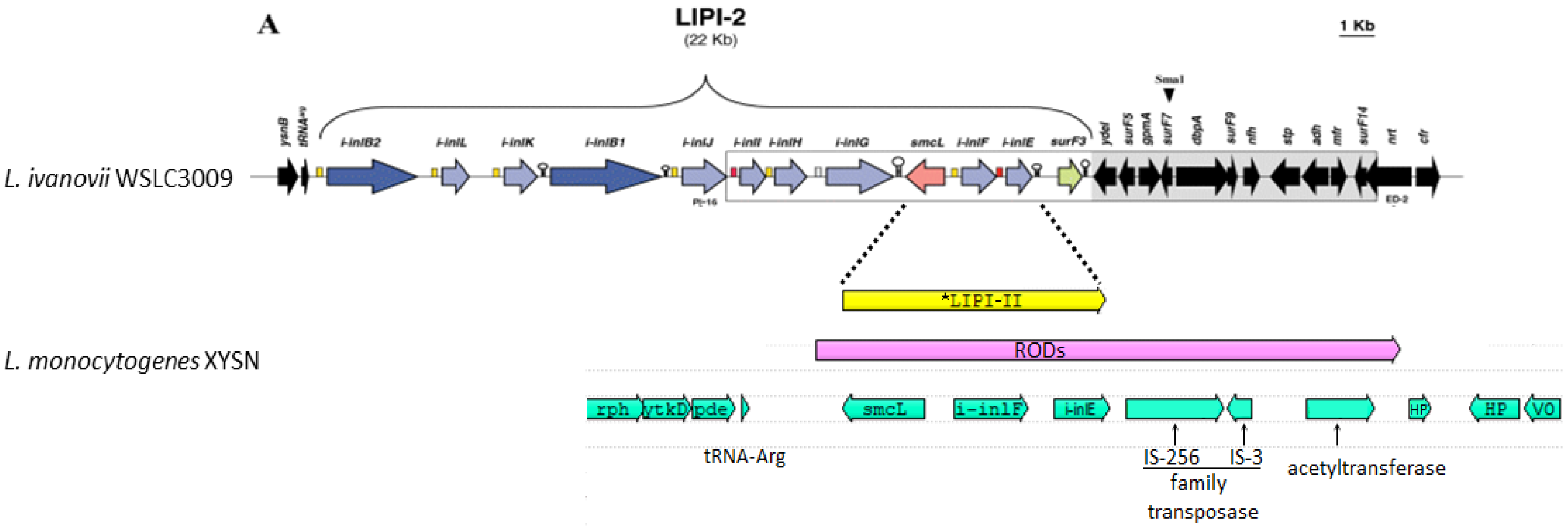

Supplementary Figure 3: Screening and identification of mutants from the transposon mutagenesis library using Caco-2 BBe infection model. (A) The invasion rate of 1127 mutants using Caco-2 BBe. A 20-fold reduction of invasion capability was observed in three of the transposon mutants. LMxysn_0462 was encoding InlA, while LMxysn_1095 and LMxysn_1098 were involved in glycosylation of wall teichoic acid (WTAs); (B) Invasion ability of mutants 1095 ∷ Tn, 1098 ∷ Tn and 0462 ∷ Tn for Caco-2 BBe cells at a MOI of 20 for 1 h. Error bars represent SD, n = 3 independent experiments. Statistical analyses were carried out by Dunnett's multiple comparisons test: ****$P < 0.0001$; (C) Multiplane confocal images of the invasive ability of XYSN and the respective transposon mutants (green) at 2 hours post entry.

A

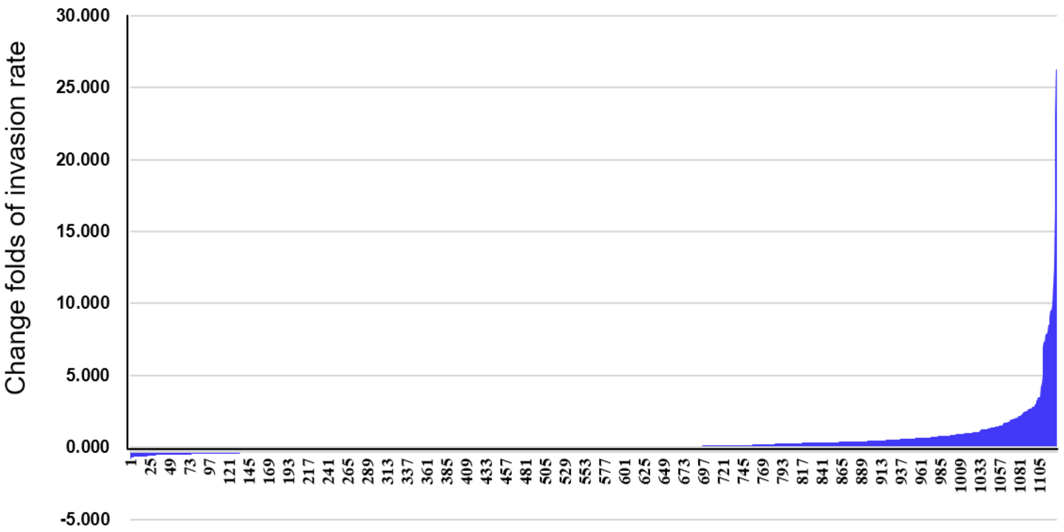

B

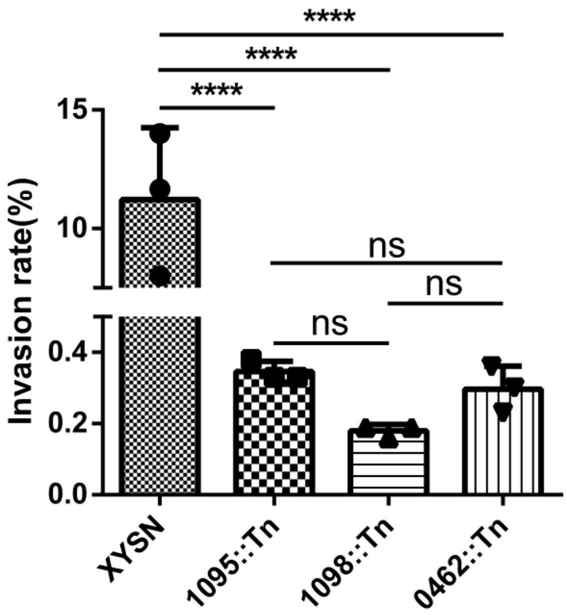

C

XYSN 1095::Tn 1098::Tn

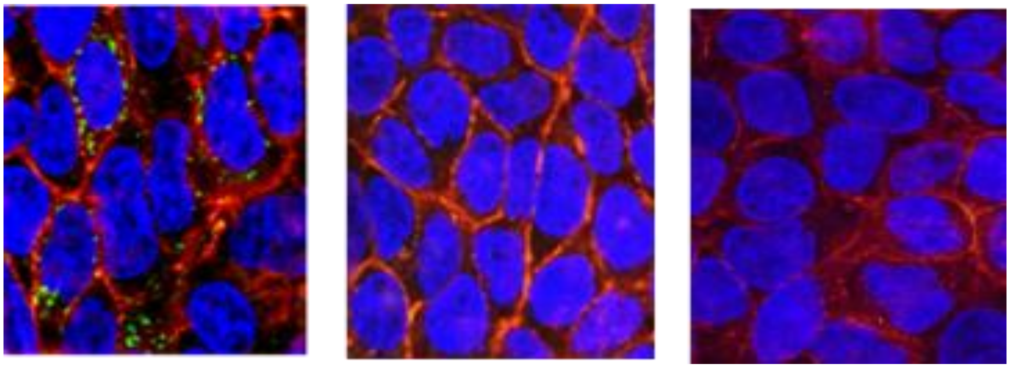

Supplementary Figure 4 : Confocal images of the adhesion capabilities of XYSN, Δ1095, Δ1095::1095 in Caco-2 BBe cells. The bacteria were GFP- labelled (green), the actin cytoskeleton was stained by phalloidin (red), and DNA was stained by DAPI (blue) following fixation and permeabilization of the sample. Magnification of all images: × 1000. Scale bars, 10 μm. Different colours in the shaded pictures were obtained using depth coding and represent the depth of subcellular structure of both bacteria and Caco-2 BBe from the surface of the slide. The experiment was repeated three times.

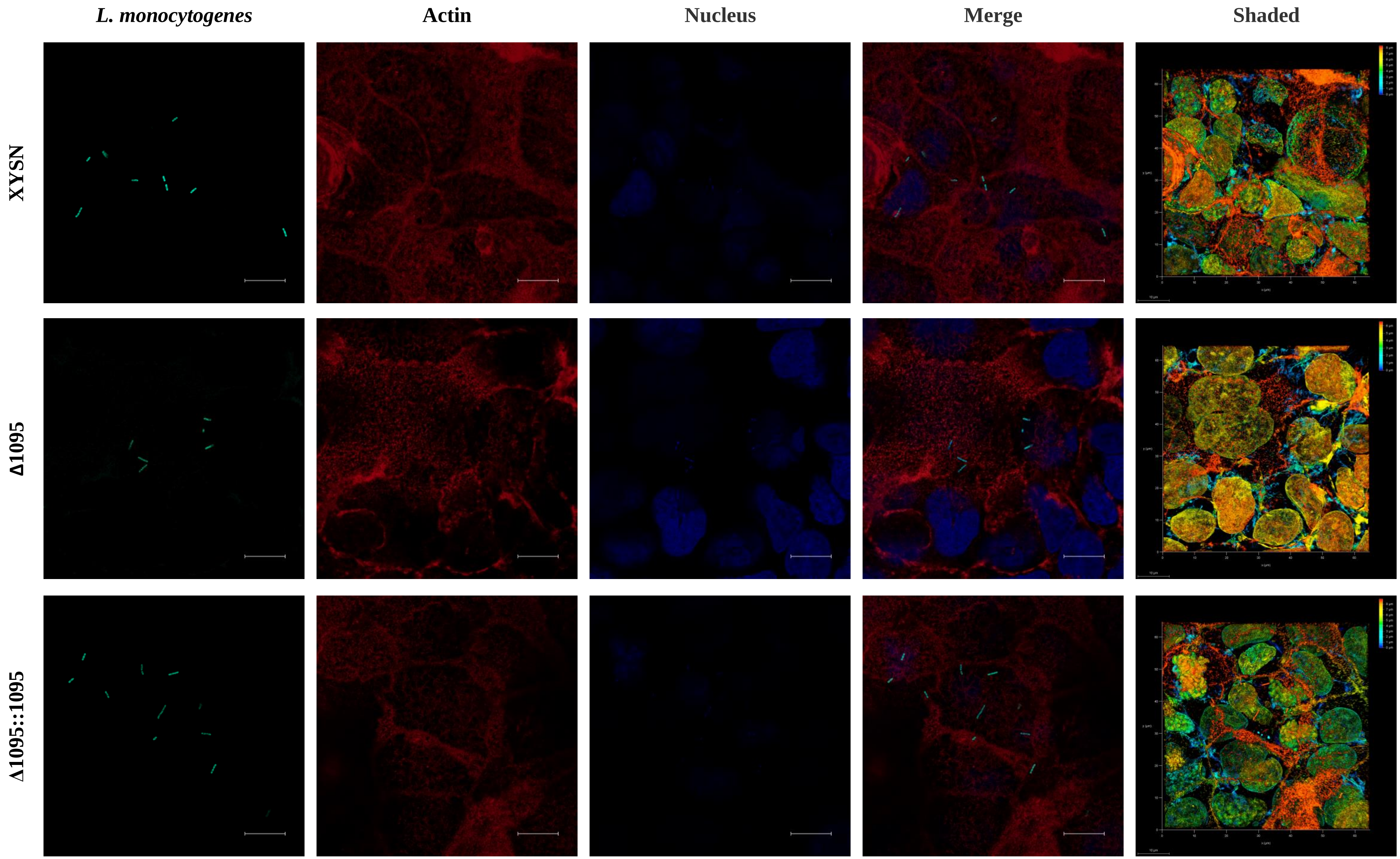

Supplementary Figure 5: LMxysn_1095 is required for agglutination with serogroup 4 anti-serum. Images of agglutination patterns obtained with XYSN, its isogenic Δ1095 mutant and the complemented strain Δ1095∷1095.

XYSN **Δ1095** **Δ1095::1095**

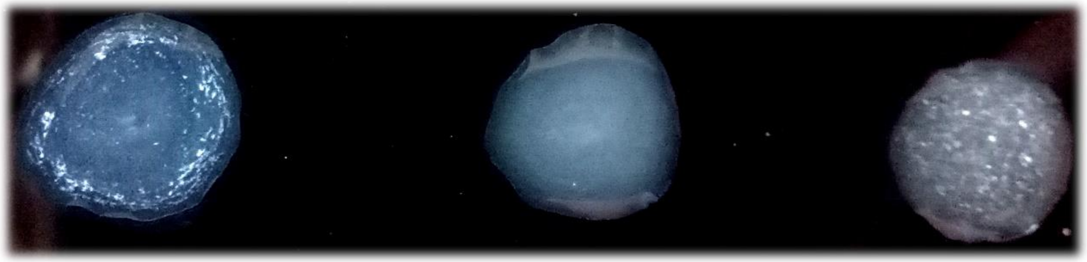

Supplementary Figure 6: Hemolytic activities of the NTSN (Lineage I), EGD-e (Lineage II) and XYSN (Hybrid Sublineage II). Culture supernatants were serially diluted in two-fold steps, added to 1% sheep blood cells and incubated at 37°C for 1 h. Microtiter wells were observed following centrifugation to pellet erythrocytes. Hemolytic activities can be directly read by visual inspection.

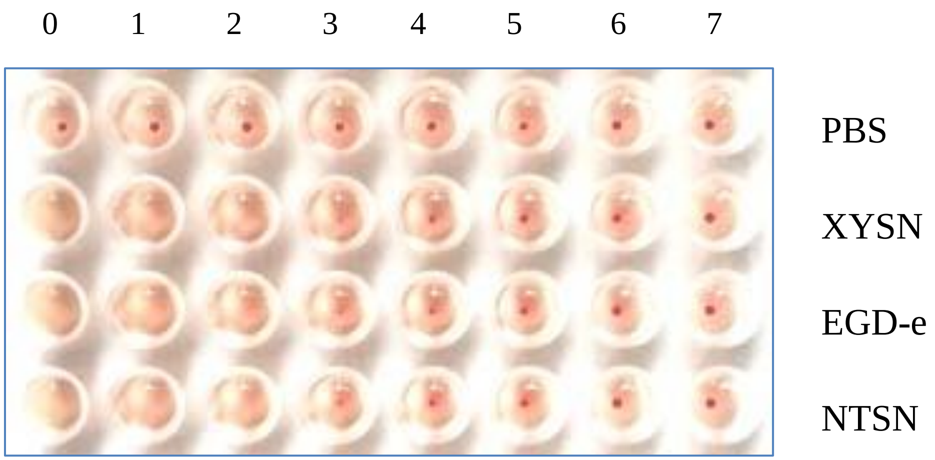

Supplementary Figure 7: *In silico* comparison of the rhamnose-operon region in lineage I-IV, lineage V (XYSN) and *L. seeligeri* SLCC 3954. The rhamnose-encoding operon (lmo2846-lmo2851) was absent in lineage IV and **HSL-II**. Comparative analysis of the same region in rhamnose-negative *L. seeligeri* genome reveals its similarity to XYSN.

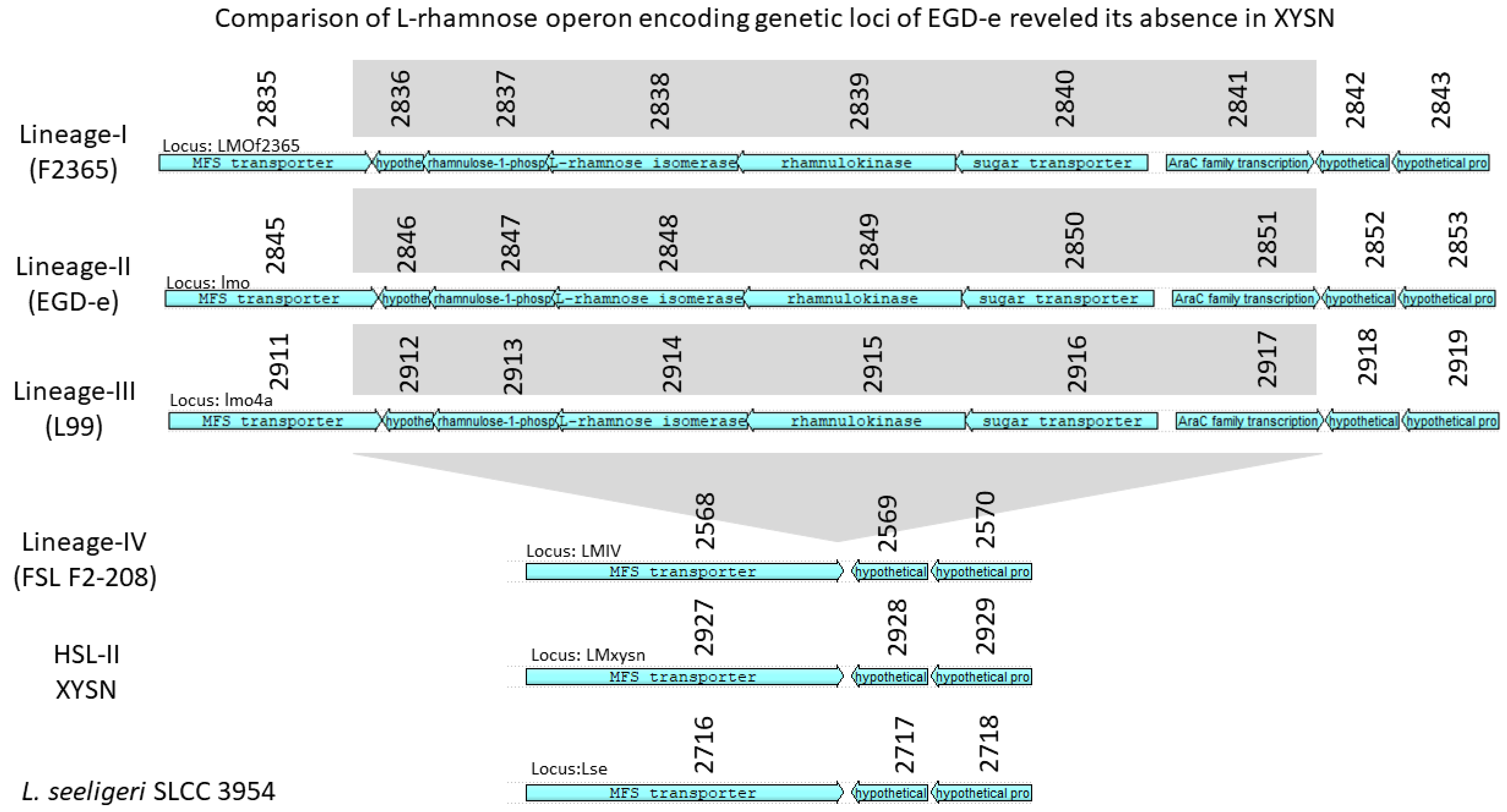

Supplementary Figure 8: Comparison of loci used for PCR-based serogrouping in the *L. monocytogenes* strains CLIP 80459 and EGD-e and their comparison with the respective genomic regions in XYSN. Three genes (orf2819, lmo1118 and lmo0737) that are part of the five-gene based serogrouping scheme of Lm are absent in XYSN[1].

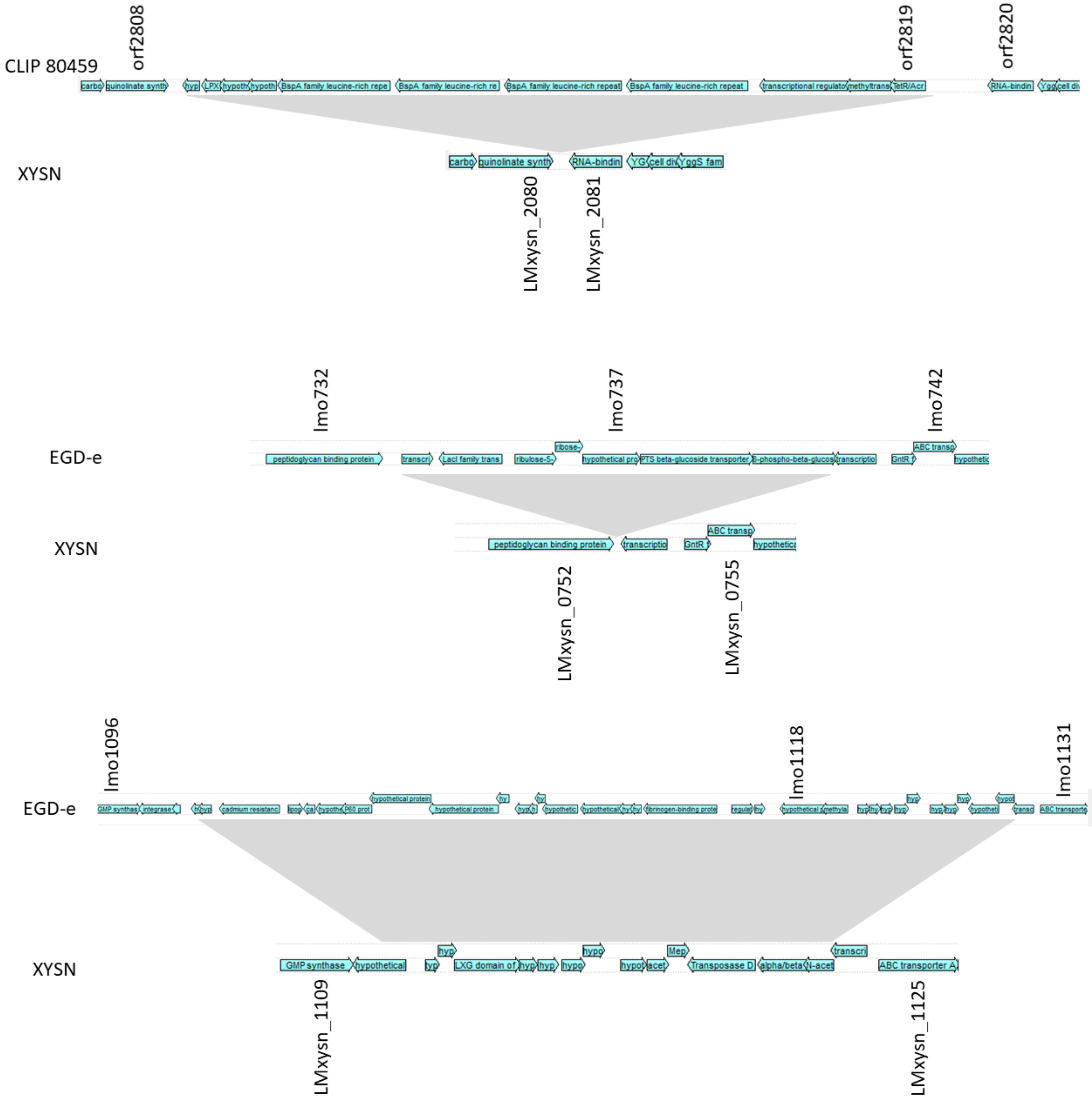

Supplementary Figure 9: Wall teichoic acid (WTA)-associated genetic cluster of XYSN and other serotypes of *Listeria*. The genetic loci and structure of WTA (as described by Shen et al., 2017)[2] are compared to highlight major differences. WTA-associated loci are depicted with orange arrows while black arrows indicate commonly occurring upstream and downstream genes. The vertical gray to black shade depicts the respective amino acid sequence homology (90-100%) (90% coverage and 90% identity). Comparative analysis revealed the genetic loci of XYSN to be closely related to that of 4b, 4e, 4d and 4f. The UPLC-MS based study of WTA supports this observation and show that the WTA of XYSN is structurally related to 4b expect that its *N*-acetylglucosamine is decorated only with galactose. The AA-sequences of WTA gene clusters of different serotype strains were compared using Easyfig[4]. Serotype 4f and 4g initially allocated to *L. monocytogenes* (Seeliger and Hohne, 1979)[3] but latter these strains were revealed to be of *L. innocua*. Therefore, to distinguish the serotype of lineage HSL- II isolates, a novel designation viz. '4h' was allocated.

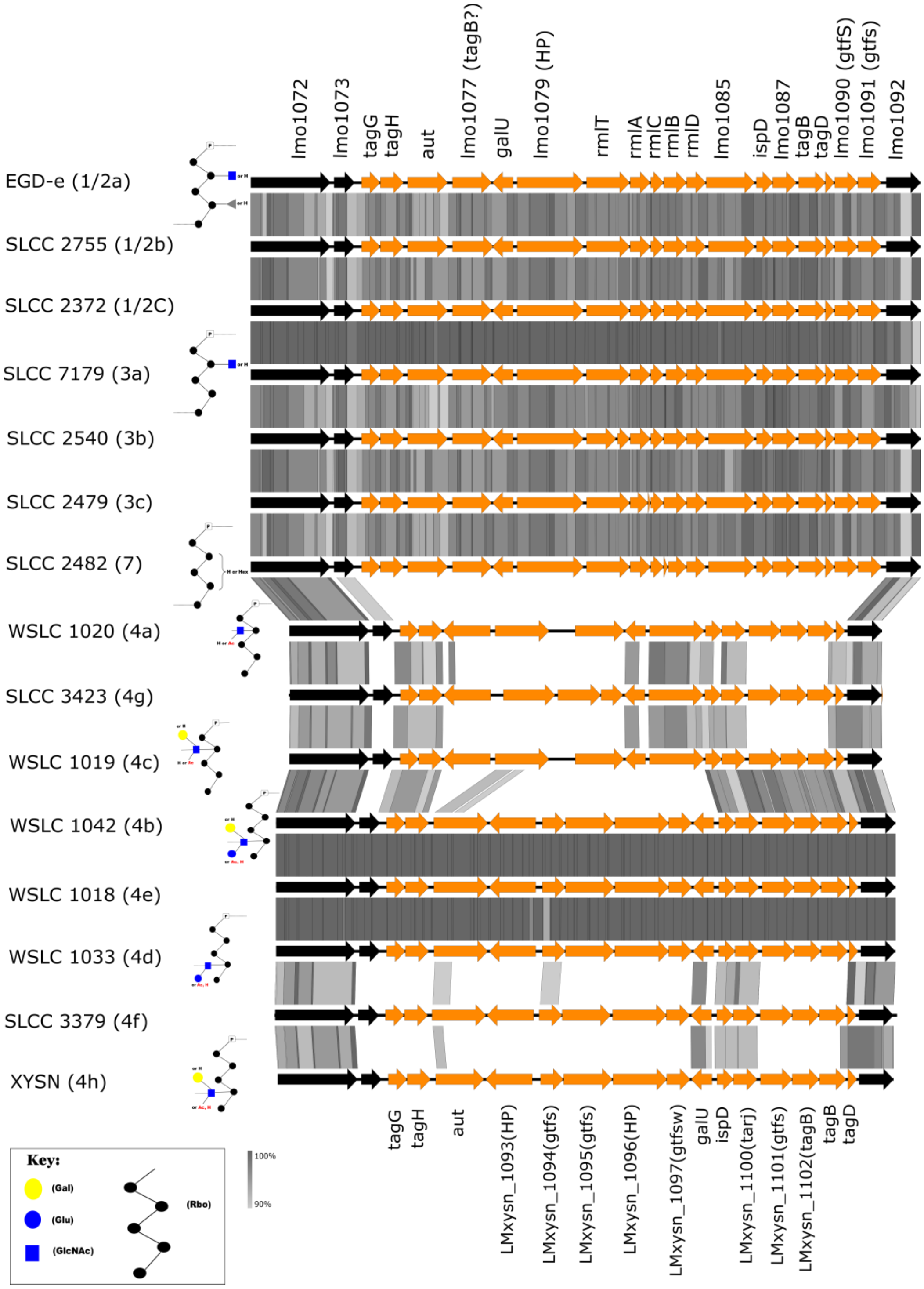

Supplementary Figure 10: Distribution of wall teichoic acid decorating genes of *L. monocytogenes* XYSN (LMxysn_1092-1104) in other lineages of *L. monocytogenes* and other *Listeria* species. Left side: A UPGMA dendrogram based on average nucleotide identity of *Listeria* species. Right side: The BLAST Score Ratio (BSR) compared to the *L. monocytogenes* XYSN, from yellow (identical gene) to blue (similar gene not present). Note, that except for *L. monocytogenes* EGD-e (lineage II), other lineages- I, III and IV, and to lesser extent to a *L. marthi* exhibits nucleotide variants of LMxysn_1092-1104. The BSR values were calculated by LS-BSR pipeline[5] and visualized in Phandango webserver[6]. Key: Lm – *L. monocytogenes.*

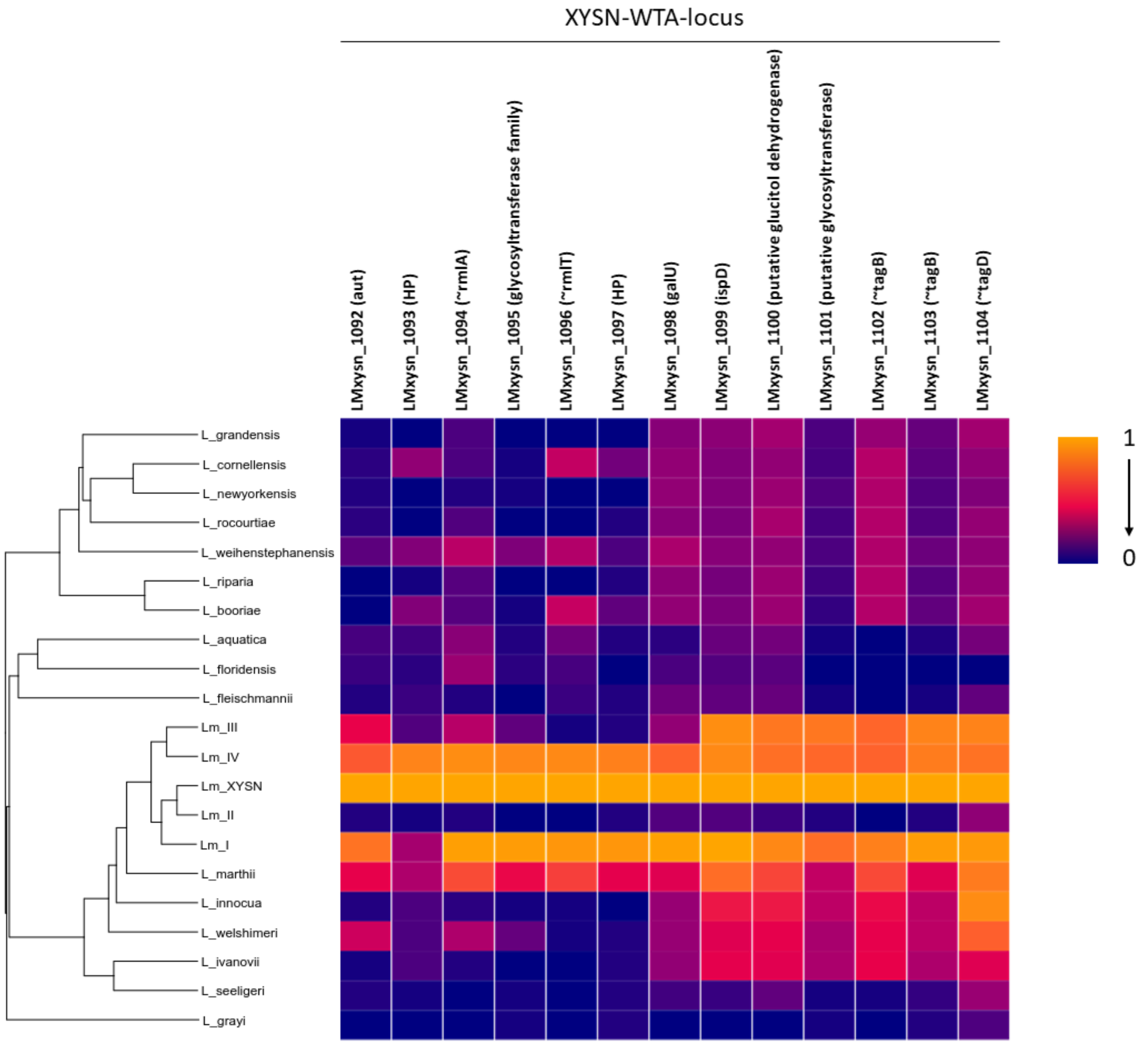

Supplementary Figure 11: Distribution of wall teichoic acid decorating genes of *L. monocytogenes* EGD-e (lmo1076-89) in other lineages of *L. monocytogenes* and other *Listeria* species. Left side: A UPGMA dendrogram based on average nucleotide identity of *Listeria* species. Right side: The BLAST Score Ratio (BSR) compared to the *L. monocytogenes* EGD-e, yellow (identical gene) ranged to blue similar (gene not present). Note, only *L. seeligeri* show nucleotide variants of lmo1076-89 (0.5-0.6 BSR). The BSR values were calculated by LS-BSR pipeline[5] and visualized in Phandango webserver[6]. Key: Lm – *L. monocytogenes*.

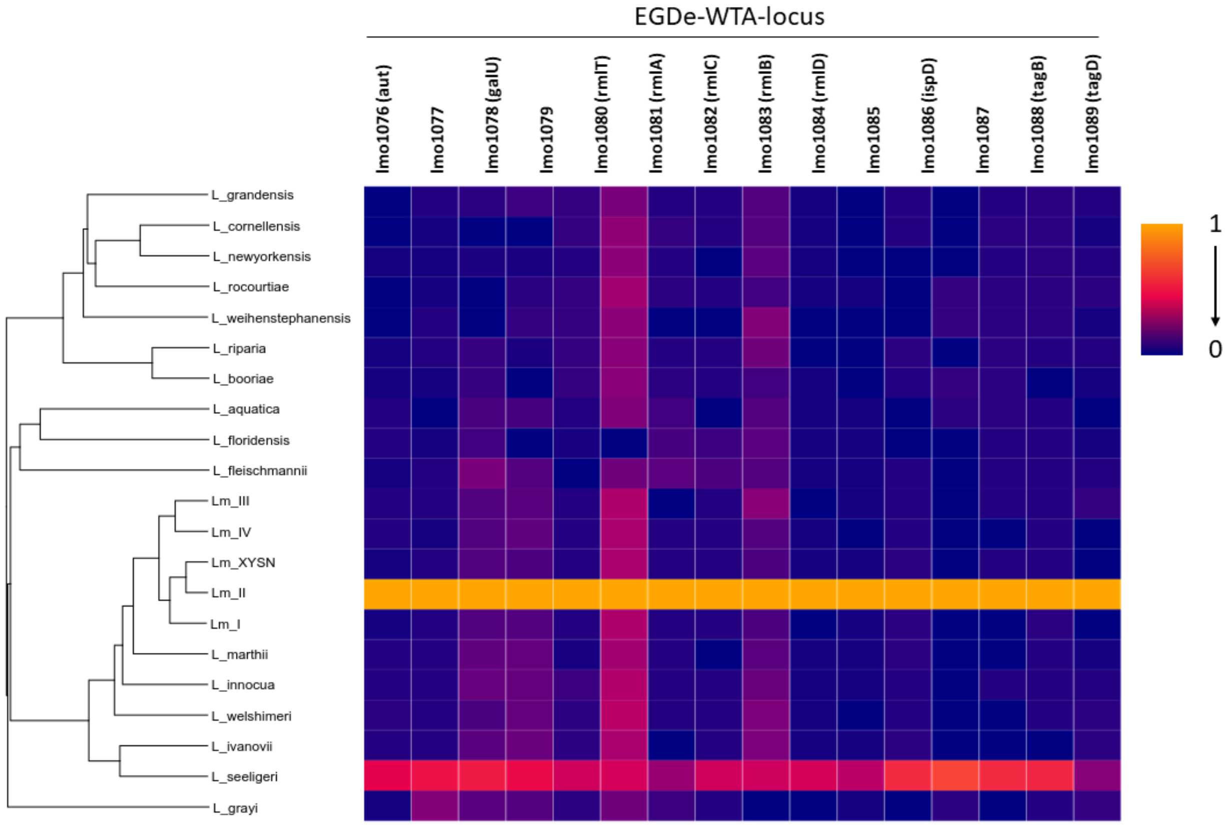

Supplementary Table 1: Bacterial strains and plasmids used in this study

Strain	Relevant characteristics	Reference/Source
L. monocytogenes XYSN	Wild type, serotypr 4h, CC33	This laboratory
L. monocytogenes 15LG	Wild type, serotypr 4h, CC33	This laboratory
L. monocytogenes 16E	Wild type, serotypr 4h, CC33	This laboratory
L. monocytogenes EGD-e	Wild type, serovar 1/2a, ST35	(Chakraborty et. al., 1992)
L. monocytogenes NTSN	Wild type, serotype 4b, CC1	This laboratory
L. monocytogenes LM11-00412	Wild type, serotype 4b, CC1	Gifted by Prof. Lecuit
L. monocytogenes LM13-00344	Wild type, serotype 4b, CC4	Gifted by Prof. Lecuit
L. monocytogenes LM6-01023	Wild type, serotype 4b, CC6	Gifted by Prof. Lecuit
L. ivanovii ZJU	Wild type, serotype 5	Gifted by Prof. Weihuan Fang
L. monocytogenes 1042	Wild type, serotype 4b	Preserved in laboratory of Food Microbiology, ETH Zurich
Rhodococcus equi	Wild type	China General Microbiological Culture Collection Center
Escherichia coli (DH5α)	Commercial strain used for cloning	Takara Biotechnology (Dalian) Co., Ltd.
pAUL-A	Em^R	(Chakraborty et. al., 1992)
pIMK-2	Km^R	Gifted by Ian R. Monk
pERL3-GFP	Em^R	(Chakraborty et. al., 2011)
TnYLB-1	Amp^R, Km^R, Spe^R	(Fan H et. al., 2016)

Supplementary Table 2: Bacterial strains constructed in this study

Strain	Relevant characteristics	Reference/Source
Δ1095	deletion *Lm*_xysn $1095_{564-1791}$	This study
Δ1095::1095	recombinant$1095_{564-1791}$ in XYSNΔ1095	This study
XYSN::*gfp*		This study
Δ1095:: *gfp*		This study
Δ1095::1095:: *gfp*		This study
Δ*smcL*	*smcL* absent in Lm XYSN	This study
Δ*hly*	*hly* absent in Lm XYSN	This study
Δ*smcL*Δ*hly*	*smcL* and *hly* absent in Lm XYSN	This study
NTSN∷*smcL*	recombinant *smcL* in the genome	This study
EGD-e∷ *smcL*	recombinant *smcL* in the genome	This study
1095∷TnYLB-1	43-bp length fragment inserted at the nucleotide site between 433 and 434 in 1095 gene	This study
1098∷TnYLB-1	55-bp length fragment inserted at the nucleotide site between 105 and 106 in 1098 gene	This study
0462∷TnYLB-1	55-bp length fragment inserted at the nucleotide site between 924 and 925 in 0462 gene	This study

Supplementary Table 3: Primers used to amplify genes

**primers**	**Sequence**	**length**
*1095*P1	5’-CGACGGCCAGTGAATTCGAATACATTGGAAAAATCTTCAAAC-3	993bp
*1095*P2	5’-ACTCTCCCTAATTCAAAATATATGTAAAGTCATAACTCTCTGCGATTGG-3	
*1095*P3	5’-CCAATCGCAGAGAGTTATGACTTTACATATATTTTGAATTAGGGAGAGT-3	903bp
*1095*P4	5’-TGCAGGTCGACTCTAGAGTTTTCACTTTTCTTACTTTCAATA-3	
*1095*W1	5’-TTATGTTTGTAGATGATGGAAGT-3	2596/3825bp
*1095*W2	5’-CCAAAGCTCAACACTAGATACTC-3	
H*1095*P1	5’-TGTAAAACGACGGCCAGTGAATTCTAGATTGAATACAAAAGGAGATGGTG-3	1930bp
H*1095*P2	5’-ATAGACTCTCCCTAATTCAAAAAATGTGTCATCTATTATCTCCAGTTTTCCTTC-3	
H*1095*P3	5’-GAAGGAAAACTGGAGATAATAGATGACACATTTTTTGAATTAGGGAGAGTCTAT-3	903bp
H*1095*P4	5’-GCATGCCTGCAGGTCGACTCTAGAGTTTTCACTTTTCTTACTTTCAATA-3	
H*1095*W1	5’-CAGCGAGAGATAGGTGTAAAGAGAG-3	3094/1865bp
H*1095*W2	5’-ATGTTTAGGAAGTCATCTGATTTGG-3	
hly P1	5’-CGGCCAGTGAATTCGAGCTCGGTACCCTCGTGTCAGTTCTGGGAGTAGT -3	786bp
hly P2	5’-TCCTAATTTTTATTGCTTTTAAAAGGGTTTCACTCTCCTTCTA -3	
hly P3	5’-TAGAAGGAGAGTGAAACCCTTTTAAAAGCAATAAAAATTAGGA -3	785bp
hly P4	5’-TCGACTCTAGAGGATCCCCGGGTACCTTTCTTTCACTGGATTTCCGATA -3	
hly wP1	5’- TCGGACCATTGTAGTCATCTTG -3	3397(1807)bp
hly wP2	5’- TCCAACTCGCAACGTCCG -3	
hly nP1	5’- CACCACCAGCATCTCCGC -3	860bp
hly nP2	5’- TTTCCCACTTACGGCAGC -3	
smcL P1	5’- CTAGTCTAGAGGACATTTCATTACATTTTTGGT -3	806bp
smcL P2	5’-TTCATTAAAAATGATGGAGGAAATAAAACAGCTACAATTTTA -3	
smcL P3	5’-TAAAATTGTAGCTGTTTTATTTCCTCCATCATTTTTAATGAA -3	759bp
smcL P4	5’-ACGCGTCGACATTATCCAAACTGAGTATCGCCA -3	

*smcL*wP1	5’-TGTAACACCCAATCGGCAGT -3	1809(801)bp
*smcL*wP2	5’-TAATTGGTAGCGGCTGTGTA -3	
*smcL*nP1	5’-ATGAATGTGGAAGTGCGTGAT -3	845bp
*smcL*nP2	5’-CGGTGCTTTCATTTTTTTACTCTT -3	
smcL pimk1	5’-GGTTAAAAAATGTAGAAGGAGAGTGAAACCCTGCAGATGGAAAAATTTAAAATTATAAAAAC -3	1076bp
smcL pimk2	5’-AACAAAAGCTGGGTACCGGGCCCCCCCTCGAGTTAGTTATTATCAGTAAAACCAAC -3	

Supplementary Table 4: Accession numbers of the closed genomes of 144 *L. monocytogenes* strains used in this study.

Accession number	Lineage
NZ_CP007462	I
NZ_CP008821	I
NZ_CP019616	I
NC_017728	I
NC_012488	I
NC_018642	I
NZ_CP011004	I
NC_018584	I
NZ_CP013288	I
NZ_CP018149	I
NZ_CP019619	I
NZ_CP013289	I
NZ_CP018148	I
NZ_CM001159	I
NZ_CP012021	I
NZ_CP015508	I
NC_021827	I
NC_021830	I
NC_021840	I
NC_021839	I
NZ_CP019625	I
NZ_CP007600	I
NZ_CP010346	I
NC_002973	I
NZ_CP009897	I
NZ_CP013285	I
NC_018585	I
NZ_CP007210	I
NZ_CP007167	I
NZ_CP007525	I
NZ_CP006874	I
NZ_CP007526	I
NC_021825	I
NZ_CP007492	I
NC_019556	I
NZ_CP019620	I
NZ_CP007459	I
NZ_CP007461	I
NZ_CP007460	I
NZ_CP020774	I
NZ_CP019615	I
NZ_CP015593	I
NZ_CP007168	I
NZ_CP007169	I
NZ_CP016213	I
NZ_CP011398	I
NZ_CP022020	I
NZ_CP023050	I

NZ_CP023052	I
NZ_CP014252	I
NC_021824	I
NZ_CP014250	I
NZ_CP019624	I
NZ_CP019622	I
NZ_CP007686	I
NC_018586	I
NZ_CP016629	I
NC_018591	I
NC_021826	I
NC_018587	I
NC_021838	I
NC_017547	II
NC_021823	II
NZ_CP020827	II
NZ_CP020830	II
NZ_CP013723	II
NZ_CM003382	II
NZ_CP011345	II
NC_003210	II
NZ_CP023754	II
NZ_CP023752	II
NZ_CP007196	II
NZ_CP007195	II
NZ_CP007194	II
NC_018589	II
NC_018588	II
NC_017546	II
NZ_CP020832	II
NZ_CP007687	II
NZ_CP019614	II
NZ_CP011397	II
NZ_CP007198	II
NZ_CP007197	II
NZ_CP013722	II
NZ_CP020828	II
NZ_CP019623	II
NZ_CP020831	II
NC_021837	II
NZ_CP007200	II
NZ_CP007199	II
NZ_CP007689	II
NZ_CP007685	II
NC_017544	II
NZ_CP007171	II
NZ_CP007170	II
NZ_CP007688	II
NZ_CP007684	II
NC_018592	II
NZ_CP007160	II
NC_022568	II
NZ_CP014261	II
NZ_CP014790	II

NZ_CP020833	II
NZ_CP019617	II
NC_017545	II
NC_018593	II
NZ_CP019621	II
NZ_CP009258	II
NZ_CP018685	II
NZ_CP007017	II
NZ_CP006861	II
NZ_CP007018	II
NZ_CP008837	II
NZ_CP019170	II
NC_013768	II
NZ_CP019164	II
NZ_CP007007	II
NZ_CP007011	II
NZ_CP019618	II
NZ_CP006862	II
NZ_CP007019	II
NZ_CP019167	II
NZ_CP007009	II
NZ_CP008836	II
NZ_CP006858	II
NZ_CP006859	II
NZ_CP006860	II
NZ_CP007010	II
NZ_CP007008	II
NZ_CP007021	II
NC_013766	II
NZ_CP019165	II
NZ_CP007020	II
NZ_CP013919	II
NZ_CP013724	II
NZ_CP009242	III
NC_017529	III
NC_011660	III
NC_017537	III
NC_018590	III
NZ_CP013287	III
NZ_CP013286	III
NZ_CM001469	IV
NZ_CM001046	IV

Supplementary Table 5: Slide agglutination patterns of *Listeria* strains using *Listeria* O and H antisera

Strains	O antigen								H antigen			
	I	I/II	IV	V/VI	VI	VII	VIII	IX	H-A	H-A/B	H-C	H-D
XYSN	-	-	-	+	+	-	-	-	+	+	-	-
15LG	-	-	-	+	+	-	-	-	+	+	-	-
16E	-	-	-	+	+	-	-	-	+	+	-	-
NTSN	-	-	-	+	+	-	-	-	+	+	+	-

Supplementary Table 6: Antimicrobial susceptibility testing

	AMP	CHL	CIP	CLI	ERY	GEN	IPM	PEN	RIF	STR	TET	SXT	VAN
XYSN	34	30	25	16	37	28	43	34	27	17	32	36	26
Sensitivity	S	S	S	I	S	S	S	S	S	S	S	S	S

Note: S: Susceptibile; R: Resistant; I: Intermediate; Unit of measurement (millimeter, mm); Abbreviations: AMP, ampicillin; PEN, penicillin G; IPM, imipenem; CIP, ciprofloxacin; TET, tetracycline; ERY, erythromycin; STR, streptomycin; GEN, gentamycin; RIF, rifampin; VAN, vancomycin; CHL, chloramphenicol; SXT, trimethoprim-sulfamethoxazole; CLI, clindamycin.

Supplementary Table 7: Virulence genes in *L. monocytogenes* XYSN determined by blastp against virulence factor database (VFDB)[7]. The additional virulence gene *smcL* identified is highlighted.

sseqid	qseqid	evalue	pident	qcovs	gene	protein	originated-from
VFG006838	Lm_XYSN_00060	0	99.77	100	agrC	hypothetical protein	*L. monocytogenes* EGD-e
VFG006834	Lm_XYSN_00061	4E-179	99.17	100	agrA	hypothetical protein	*L. monocytogenes* EGD-e
VFG000072	Lm_XYSN_00220	4E-174	99.16	100	prfA	listeriolysin positive regulatory protein	*L. monocytogenes* EGD-e
VFG032323	Lm_XYSN_00221	0	96.21	100	plcA	phosphatidylinositol-specific phospholipase C	*L. monocytogenes* SLCC2376
VFG032270	Lm_XYSN_00222	0	97.73	100	hly	listeriolysin O	*L. monocytogenes* SLCC2376
VFG032285	Lm_XYSN_00223	0	96.86	100	mpl	zinc metalloproteinase	*L. monocytogenes* SLCC5850
VFG000068	Lm_XYSN_00224	0	83.91	99	actA	actin-assembly inducing protein precursor	*L. monocytogenes* EGD-e
VFG032340	Lm_XYSN_00225	0	97.58	100	plcB	phosphatidylcholine-specific phospholipase C	*L. monocytogenes* SLCC7179
VFG000079	Lm_XYSN_00268	0	100	100	clpC	endopeptidase Clp ATP-binding chain C	*L. monocytogenes* EGD-e
VFG033049	Lm_XYSN_00432	0	97	100	pdgA	polysaccharide deacetylase	*L. monocytogenes* Finland 1998
VFG031991	Lm_XYSN_00473	0	97	100	inlA	internalin A	*L. monocytogenes* SLCC7179
VFG032025	Lm_XYSN_00474	0	95.24	100	inlB	internalin B	*L. monocytogenes* J1816
VFG032985	Lm_XYSN_00477	6E-141	97.56	100	lntA	hypothetical protein	*L. monocytogenes* Finland 1998
VFG032102	Lm_XYSN_00610	0	99.58	100	iap/cwhA	p60	*L. monocytogenes* J0161
VFG043247	Lm_XYSN_00712	0	98.43	100	fliP	flagellar biosynthesis protein FliP	*L. monocytogenes* EGD-e
VFG043249	Lm_XYSN_00714	3E-177	100	100	fliR	flagellar biosynthesis protein FliR	*L. monocytogenes* EGD-e
VFG043250	Lm_XYSN_00715	0	99.71	100	flhB	flagellar biosynthesis protein FlhB	*L. monocytogenes* EGD-e
VFG043251	Lm_XYSN_00716	0	99.86	100	flhA	flagellar biosynthesis protein FlhA	*L. monocytogenes* EGD-e
VFG043252	Lm_XYSN_00717	0	100	100	flhF	flagellar biosynthesis regulator FlhF	*L. monocytogenes* EGD-e
VFG043253	Lm_XYSN_00718	0	99.23	100	flgG	flagellar basal body rod protein FlgG	*L. monocytogenes* EGD-e
VFG043254	Lm_XYSN_00719	0	100	100	cheR	hypothetical protein	*L. monocytogenes* EGD-e
VFG043255	Lm_XYSN_00721	0	100	100	motA	flagellar motor protein MotA	*L. monocytogenes* EGD-e
VFG043256	Lm_XYSN_00722	0	98.91	100	motB	hypothetical protein	*L. monocytogenes* EGD-e
VFG043257	Lm_XYSN_00725	0	100	100	cheV	hypothetical protein	*L. monocytogenes* EGD-e
VFG043258	Lm_XYSN_00726	0	100	100	flaA	flagellin	*L. monocytogenes* EGD-e
VFG006842	Lm_XYSN_00728	0	99.84	100	cheA	two-component sensor histidine kinase CheA	*L. monocytogenes* EGD-e
VFG043261	Lm_XYSN_00733	0	99.51	100	flgE	flagellar hook protein FlgE	*L. monocytogenes* EGD-e
VFG043263	Lm_XYSN_00735	0	99.7	100	fliM	flagellar motor switch protein FliM	*L. monocytogenes* EGD-e
VFG043264	Lm_XYSN_00736	0	99.61	100	lmo0700	flagellar motor switch protein	*L. monocytogenes* EGD-e
VFG043265	Lm_XYSN_00741	0	99.6	100	flgK	flagellar hook-associated protein FlgK	*L. monocytogenes* EGD-e
VFG043266	Lm_XYSN_00742	0	99.66	100	flgL	flagellar hook-associated protein FlgL	*L. monocytogenes* EGD-e
VFG043267	Lm_XYSN_00743	0	99.07	100	fliD	flagellar capping protein	*L. monocytogenes* EGD-e
VFG043272	Lm_XYSN_00749	0	99.45	100	fliF	flagellar MS-ring protein	*L. monocytogenes* EGD-e

VFG043273	Lm_XYSN_00750	0	99.73	100	fliG	flagellar motor switch protein G	*L. monocytogenes* EGD-e
VFG043274	Lm_XYSN_00751	3E-167	100	100	fliH	flagellar assembly protein H	*L. monocytogenes* EGD-e
VFG043275	Lm_XYSN_00752	0	99.31	100	fliI	flagellum-specific ATP synthase	*L. monocytogenes* EGD-e
VFG032167	Lm_XYSN_00871	0	99.78	100	hpt	sugar phosphate transporter	*L. monocytogenes* Finland 1998
VFG006798	Lm_XYSN_00967	1E-161	100	100	srtA	hypothetical protein	*L. monocytogenes* EGD-e
VFG002158	Lm_XYSN_00969	0	100	100	lplA1	lipoate protein ligase	*L. monocytogenes* EGD-e
VFG032812	Lm_XYSN_01013	0	100	100	dltA	D-alanine--poly(phosphoribitol	*L. monocytogenes* 10403S
VFG000080	Lm_XYSN_01037	0	99.59	100	clpE	ATP-dependent protease	*L. monocytogenes* EGD-e
VFG000674	Lm_XYSN_01259	0	94.33	100	smcL	sphingomyelinase-c	*L. ivanovii* str. ATCC 19119
VFG033003	Lm_XYSN_01319	0	99.68	100	oatA	O-acetyltransferase	*L. monocytogenes* SLCC2540
VFG006826	Lm_XYSN_01407	5E-162	100	100	lisR	two-component response regulator	*L. monocytogenes* EGD-e
VFG006830	Lm_XYSN_01408	0	99.79	100	lisK	two-component sensor histidine kinase	*L. monocytogenes* EGD-e
VFG031937	Lm_XYSN_01680	0	100	100	lap	bifunctional aldehyde-alcohol dehydrogenase	*L. monocytogenes* SLCC5850
VFG032574	Lm_XYSN_01823	0	99.71	100	virS	hypothetical protein	*L. monocytogenes* 08-5578
VFG006818	Lm_XYSN_01827	2E-165	100	100	virR	hypothetical protein	*L. monocytogenes* EGD-e
VFG032911	Lm_XYSN_01871	0	97.98	100	inlC	internalin family protein	*L. monocytogenes* F2365
VFG006794	Lm_XYSN_01913	0	100	100	stp	hypothetical protein	*L. monocytogenes* EGD-e
VFG002160	Lm_XYSN_01921	0	99.82	100	fbpA	fibronectin-binding protein	*L. monocytogenes* EGD-e
VFG002161	Lm_XYSN_01937	1E-107	100	100	lspA	signal peptidase II	*L. monocytogenes* EGD-e
VFG032074	Lm_XYSN_01940	0	99.68	100	lpeA	manganese-binding lipoprotein mntA	*L. monocytogenes* Finland 1998
VFG002162	Lm_XYSN_02160	0	99.69	100	bsh	bile salt hydrolase	*L. monocytogenes* EGD-e
VFG032414	Lm_XYSN_02290	9E-180	98.37	100	srtB	hypothetical protein	*L. monocytogenes* 08-5578
VFG001090	Lm_XYSN_02294	0	100	100	hbp2	hemin/hemoglobin-binding protein 2	*L. monocytogenes* EGD-e
VFG045468	Lm_XYSN_02295	3E-148	99.52	100	hdp1	Haemoglobin binding protein 1	*L. monocytogenes* EGD-e
VFG006782	Lm_XYSN_02305	0	100	100	oppA	hypothetical protein	*L. monocytogenes* EGD-e
VFG032879	Lm_XYSN_02328	0	99.66	100	prsA2	protein export protein	*L. monocytogenes* serotype. F2365
VFG000077	Lm_XYSN_02543	1E-143	100	100	clpP	ATP-dependent Clp protease proteolytic subunit	*L. monocytogenes* EGD-e
Lmo2470	Lm_XYSN_02543	0	91	100	inlP	Internalin P	*L. monocytogenes* EGD-e
VFG006806	Lm_XYSN_02557	0	100	100	lgt	hypothetical protein	*L. monocytogenes* EGD-e
VFG032863	Lm_XYSN_02624	8E-101	100	100	gtcA	cell wall teichoic acid glycosylation protein gtcA	*L. monocytogenes* J1816
VFG031981	Lm_XYSN_02970	0	98.71	100	inlJ	internalin	*L. monocytogenes* J0161

Supplementary Table 8: Pan genome comparison of HSL-Ⅱ strains (XYSN, 15LG and 16E) using Roary[8].

Annotation	15LG	16E	XYSN
hypothetical protein	15LG_00102	16E_00115	
hypothetical protein		16E_00990	Lm_XYSN_00980
PTS system glucose-specific EIICBA component		16E_01086	Lm_XYSN_01076
hypothetical protein		16E_00182	Lm_XYSN_00166
hypothetical protein		16E_00183	Lm_XYSN_00167
Bacillus transposase protein	15LG_00109		Lm_XYSN_00095
hypothetical protein		16E_02992	Lm_XYSN_02170
hypothetical protein	15LG_00234	16E_00257	
hypothetical protein	15LG_02923	16E_02947	
hypothetical protein	15LG_02931	16E_02980	
Transposase	15LG_02924		Lm_XYSN_01318
hypothetical protein		16E_02991	Lm_XYSN_02171
hypothetical protein	15LG_00915		Lm_XYSN_00875
hypothetical protein	15LG_01661	16E_01636	
hypothetical protein	15LG_02925		Lm_XYSN_01317
hypothetical protein		16E_00415	Lm_XYSN_00398
hypothetical protein		16E_00186	Lm_XYSN_00498
hypothetical protein	15LG_00387		Lm_XYSN_00397
hypothetical protein		16E_00474	Lm_XYSN_00457
hypothetical protein		16E_00476	Lm_XYSN_00459
hypothetical protein		16E_00675	Lm_XYSN_00670
hypothetical protein		16E_00676	Lm_XYSN_00671
hypothetical protein		16E_00677	Lm_XYSN_00672
Matrixin		16E_00801	Lm_XYSN_00797
hypothetical protein		16E_00802	Lm_XYSN_00798
hypothetical protein		16E_00805	Lm_XYSN_00800
VanZ like family protein		16E_00806	Lm_XYSN_00801
hypothetical protein		16E_00807	Lm_XYSN_00802
Internalin B precursor		16E_02626	Lm_XYSN_02668
preprotein translocase subunit SecB		16E_02627	Lm_XYSN_02669
hypothetical protein		16E_02628	Lm_XYSN_02670
hypothetical protein		16E_02629	Lm_XYSN_02671
hypothetical protein		16E_02636	Lm_XYSN_02678
hypothetical protein		16E_02637	Lm_XYSN_02679
hypothetical protein		16E_02638	Lm_XYSN_02680
Phage-related minor tail protein		16E_02639	Lm_XYSN_02681
hypothetical protein		16E_02640	Lm_XYSN_02682
hypothetical protein		16E_02641	Lm_XYSN_02683
Bacterial Ig-like domain (group 2)		16E_02642	Lm_XYSN_02684

hypothetical protein		16E_02643	Lm_XYSN_02685
hypothetical protein		16E_02644	Lm_XYSN_02686
hypothetical protein		16E_02645	Lm_XYSN_02687
hypothetical protein		16E_02646	Lm_XYSN_02688
hypothetical protein		16E_02647	Lm_XYSN_02689
hypothetical protein		16E_02648	Lm_XYSN_02690
hypothetical protein		16E_02649	Lm_XYSN_02691
hypothetical protein		16E_02650	Lm_XYSN_02692
Phage minor capsid protein 2		16E_02651	Lm_XYSN_02693
Phage portal protein, SPP1 Gp6-like		16E_02652	Lm_XYSN_02694
Terminase-like family protein		16E_02653	Lm_XYSN_02695
Phage terminase small subunit		16E_02654	Lm_XYSN_02696
hypothetical protein		16E_02655	Lm_XYSN_02697
hypothetical protein		16E_02656	Lm_XYSN_02698
hypothetical protein		16E_02657	Lm_XYSN_02699
hypothetical protein		16E_02658	Lm_XYSN_02700
hypothetical protein		16E_02659	Lm_XYSN_02701
hypothetical protein		16E_02660	Lm_XYSN_02702
Single-stranded DNA-binding protein A		16E_02661	Lm_XYSN_02703
hypothetical protein		16E_02662	Lm_XYSN_02704
hypothetical protein		16E_02663	Lm_XYSN_02705
hypothetical protein		16E_02664	Lm_XYSN_02706
recombination and repair protein RecT		16E_02665	Lm_XYSN_02707
DNA replication and repair protein RecF		16E_02666	Lm_XYSN_02708
hypothetical protein		16E_02667	Lm_XYSN_02709
hypothetical protein		16E_02668	Lm_XYSN_02710
hypothetical protein		16E_02669	Lm_XYSN_02711
hypothetical protein		16E_02670	Lm_XYSN_02712
hypothetical protein		16E_02671	Lm_XYSN_02713
hypothetical protein		16E_02672	Lm_XYSN_02714
hypothetical protein		16E_02673	Lm_XYSN_02715
HTH-type transcriptional regulator Xre		16E_02674	Lm_XYSN_02716
hypothetical protein		16E_02675	Lm_XYSN_02717
hypothetical protein		16E_02676	Lm_XYSN_02718
Tyrosine recombinase XerD		16E_02677	Lm_XYSN_02719
hypothetical protein		16E_02958	Lm_XYSN_00084
hypothetical protein		16E_02962	Lm_XYSN_00461
hypothetical protein	15LG_02940	16E_02993	
Transposase, Mutator family		16E_00803	Lm_XYSN_00799
hypothetical protein	15LG_02446		Lm_XYSN_02458
hypothetical protein	15LG_02448		Lm_XYSN_02460
hypothetical protein	15LG_02447		Lm_XYSN_02459

hypothetical protein	15LG_00101	16E_00114	
hypothetical protein	15LG_00108		Lm_XYSN_00094
Putative HTH-type transcriptional regulator YwnA	15LG_00463	16E_00491	
Putative HTH-type transcriptional regulator YwnA		16E_00488	Lm_XYSN_00475
hypothetical protein	15LG_02934		
hypothetical protein		16E_00895	
Protoporphyrinogen oxidase		16E_02988	
Isocitrate dehydrogenase [NADP]			Lm_XYSN_01609
hypothetical protein	15LG_02849		
hypothetical protein	15LG_02885		
Integrase core domain protein			Lm_XYSN_00119
Transposase, Mutator family			Lm_XYSN_00171
Transposase, Mutator family			Lm_XYSN_00396
Transposase, Mutator family			Lm_XYSN_02536
hypothetical protein			Lm_XYSN_00495
tRNA(Glu)-specific nuclease WapA		16E_02961	
tRNA(Glu)-specific nuclease WapA		16E_02963	
tRNA(Glu)-specific nuclease WapA precursor			Lm_XYSN_00456
tRNA(Glu)-specific nuclease WapA precursor			Lm_XYSN_00460
hypothetical protein			Lm_XYSN_00496
tRNA3(Ser)-specific nuclease WapA		16E_00475	
hypothetical protein	15LG_00446		
hypothetical protein	15LG_00103		
hypothetical protein	15LG_00104		
hypothetical protein	15LG_00105		
hypothetical protein	15LG_00106		
hypothetical protein	15LG_00442		
hypothetical protein	15LG_00443		
hypothetical protein	15LG_00444		
Transposase from transposon Tn916	15LG_00647		
hypothetical protein	15LG_00648		
hypothetical protein	15LG_00649		
hypothetical protein	15LG_00650		
hypothetical protein	15LG_00651		
Transposase			Lm_XYSN_01631
hypothetical protein	15LG_00652		
Transposase			Lm_XYSN_01767
Transposase			Lm_XYSN_01814
hypothetical protein	15LG_00653		
hypothetical protein	15LG_00654		
hypothetical protein	15LG_00655		
hypothetical protein	15LG_00656		

hypothetical protein	15LG_00657		
hypothetical protein	15LG_00658		
hypothetical protein	15LG_00659		
hypothetical protein	15LG_00661		
Aryl-phospho-beta-D-glucosidase BglH		16E_00361	
hypothetical protein	15LG_00662		
hypothetical protein	15LG_00663		
hypothetical protein	15LG_00664		
hypothetical protein	15LG_00665		
hypothetical protein	15LG_00666		
Secreted effector protein PipB2	15LG_00667		
hypothetical protein	15LG_00668		
hypothetical protein	15LG_00669		
hypothetical protein	15LG_00671		
hypothetical protein	15LG_00672		
Aryl-phospho-beta-D-glucosidase BglH			Lm_XYSN_00345
hypothetical protein	15LG_00673		
hypothetical protein	15LG_00674		
hypothetical protein	15LG_00675		
hypothetical protein	15LG_00676		
hypothetical protein	15LG_00677		
hypothetical protein	15LG_00678		
Putative DNA repair helicase RadD	15LG_00679		
hypothetical protein	15LG_00680		
hypothetical protein	15LG_00681		
hypothetical protein	15LG_00682		
hypothetical protein	15LG_00683		
Integrase core domain protein			Lm_XYSN_00588
Integrase core domain protein			Lm_XYSN_00623
Integrase core domain protein			Lm_XYSN_01630
Integrase core domain protein			Lm_XYSN_01766
Integrase core domain protein			Lm_XYSN_01813
hypothetical protein	15LG_00684		
Integrase core domain protein			Lm_XYSN_02032
hypothetical protein	15LG_00685		
hypothetical protein	15LG_00686		
Transposase			Lm_XYSN_00355
hypothetical protein	15LG_00687		
Transposase DDE domain protein			Lm_XYSN_00181
Transposase DDE domain protein			Lm_XYSN_00650
Transposase DDE domain protein			Lm_XYSN_00708
Transposase DDE domain protein			Lm_XYSN_00944

Transposase DDE domain protein		Lm_XYSN_00950
Transposase DDE domain protein		Lm_XYSN_00964
Transposase DDE domain protein		Lm_XYSN_01145
Transposase DDE domain protein		Lm_XYSN_01151
Transposase DDE domain protein		Lm_XYSN_01360
hypothetical protein	15LG_00688	
Transposase DDE domain protein		Lm_XYSN_01409
Transposase DDE domain protein		Lm_XYSN_01531
Transposase DDE domain protein		Lm_XYSN_01562
Transposase DDE domain protein		Lm_XYSN_01591
Transposase DDE domain protein		Lm_XYSN_01668
Transposase DDE domain protein		Lm_XYSN_01867
Transposase DDE domain protein		Lm_XYSN_01873
Transposase DDE domain protein		Lm_XYSN_01923
Transposase DDE domain protein		Lm_XYSN_02042
Transposase DDE domain protein		Lm_XYSN_02068
hypothetical protein	15LG_00689	
Transposase DDE domain protein		Lm_XYSN_02086
Transposase DDE domain protein		Lm_XYSN_02157
Transposase DDE domain protein		Lm_XYSN_02185
Transposase DDE domain protein		Lm_XYSN_02238
Transposase DDE domain protein		Lm_XYSN_02479
Transposase DDE domain protein		Lm_XYSN_02736
Transposase DDE domain protein		Lm_XYSN_02992
hypothetical protein	15LG_00690	
Chromosome partition protein Smc	15LG_00691	
hypothetical protein	15LG_00692	
Integrase core domain protein		Lm_XYSN_00480
Integrase core domain protein		Lm_XYSN_01285
Integrase core domain protein		Lm_XYSN_02450
Integrase core domain protein		Lm_XYSN_02852
Pesticidal crystal protein Cry22Aa	15LG_02936	
hypothetical protein	15LG_00693	
Transposase		Lm_XYSN_01548
hypothetical protein	15LG_00694	
Transposase		Lm_XYSN_02063
hypothetical protein	15LG_00695	
hypothetical protein	15LG_00696	
Integrase core domain protein		Lm_XYSN_00354
hypothetical protein	15LG_00697	
hypothetical protein	15LG_00698	
putative protein YqbO	15LG_00699	

hypothetical protein			Lm_XYSN_00194
hypothetical protein	15LG_00700		
hypothetical protein	15LG_00701		
hypothetical protein	15LG_00708		
hypothetical protein		16E_02985	
hypothetical protein	15LG_00709		
Transposase, Mutator family			Lm_XYSN_00092
Transposase, Mutator family			Lm_XYSN_00464
hypothetical protein	15LG_00710		
Transposase, Mutator family			Lm_XYSN_00837
Transposase, Mutator family			Lm_XYSN_01262
Transposase, Mutator family			Lm_XYSN_01316
Transposase, Mutator family			Lm_XYSN_01444
Transposase, Mutator family			Lm_XYSN_01643
Transposase, Mutator family			Lm_XYSN_02169
Transposase, Mutator family			Lm_XYSN_02265
Transposase, Mutator family			Lm_XYSN_02501
hypothetical protein	15LG_00711		
hypothetical protein	15LG_00835		
Glutamyl endopeptidase	15LG_00836		
hypothetical protein	15LG_00837		
Transposase			Lm_XYSN_00244
Transposase			Lm_XYSN_00587
Transposase			Lm_XYSN_00622
Transposase			Lm_XYSN_02033
hypothetical protein	15LG_00838		
hypothetical protein	15LG_00839		
hypothetical protein	15LG_00840		
hypothetical protein	15LG_00841		
hypothetical protein	15LG_00842		
hypothetical protein	15LG_00843		
hypothetical protein	15LG_02935		
hypothetical protein		16E_00033	
Integrase core domain protein			Lm_XYSN_01547
Integrase core domain protein			Lm_XYSN_02062
putative murein peptide carboxypeptidase		16E_00043	
hypothetical protein		16E_00044	
Sugar phosphatase YidA		16E_00045	
Catabolite control protein A		16E_00046	
N-acetylmannosamine kinase		16E_00047	
Endoglucanase D		16E_00048	
PTS system oligo-beta-mannoside-specific EIIC component		16E_00049	

Glutamine--fructose-6-phosphate aminotransferase [isomerizing]		16E_00050
hypothetical protein		16E_00086
ESAT-6 secretion system extracellular protein A		16E_00095
ESAT-6 secretion accessory factor EsaA		16E_00096
hypothetical protein		16E_00097
ESAT-6 secretion accessory factor EsaB		16E_00098
ESAT-6 secretion machinery protein EssB		16E_00099
hypothetical protein		16E_00414
hypothetical protein		16E_00101
ESAT-6 secretion system extracellular protein B		16E_00102
hypothetical protein		16E_00103
hypothetical protein		16E_00104
ESAT-6 secretion machinery protein EssD		16E_00105
putative ABC transporter ATP-binding protein		16E_00149
hypothetical protein		16E_00191
hypothetical protein		16E_00192
hypothetical protein		16E_00193
hypothetical protein		16E_00194
hypothetical protein		16E_00196
hypothetical protein		16E_00197
Internalin-A		16E_00312
hypothetical protein		16E_00367
Sec-independent protein translocase protein TatCy		16E_00410
Sec-independent protein translocase protein TatAy		16E_00411
putative peptidase		16E_00412
Pesticidal crystal protein Cry22Aa		16E_00490
hypothetical protein		16E_00510
Putative trans-acting regulator		16E_00511
hypothetical protein		16E_00512
hypothetical protein	15LG_00343	
Biofilm operon icaADBC HTH-type negative transcriptional regulator IcaR		16E_00896
Quaternary ammonium compound-resistance protein SugE		16E_00897
Quaternary ammonium compound-resistance protein SugE		16E_00898
Phosphoribosylformylglycinamidine cyclo-ligase		16E_01846
Putative cysteine desulfurase NifS		16E_01847
L-aspartate oxidase		16E_01848
putative nicotinate-nucleotide pyrophosphorylase [carboxylating]		16E_01849
Maltose phosphorylase		16E_01850
hypothetical protein		16E_02612

Putative monooxygenase YcnE		16E_02613	
hypothetical protein	15LG_01882		
Transposase DDE domain protein			Lm_XYSN_02449
hypothetical protein	15LG_02157		
hypothetical protein		16E_00195	
hypothetical protein	15LG_02939		
hypothetical protein		16E_02886	
Nucleoid occlusion protein		16E_02887	
hypothetical protein		16E_00211	
hypothetical protein		16E_02946	
Deoxyguanosinetriphosphate triphosphohydrolase		16E_02968	
hypothetical protein		16E_02972	
Phosphate acetyltransferase		16E_02982	
Adenosyl-chloride synthase		16E_02983	
hypothetical protein		16E_02984	
Holo-[acyl-carrier-protein] synthase		16E_02987	
hypothetical protein		16E_02994	
hypothetical protein		16E_01531	
Actin assembly-inducing protein		16E_02996	
Zinc metalloproteinase		16E_02997	
hypothetical protein			Lm_XYSN_00245
hypothetical protein			Lm_XYSN_00246
hypothetical protein			Lm_XYSN_01785
Integrase core domain protein			Lm_XYSN_02168
hypothetical protein	15LG_01295		
hypothetical protein		16E_00413	
hypothetical protein		16E_00804	
CRISPR-associated endonuclease Cas9		16E_02970	
hypothetical protein		16E_00185	
CRISPR-associated endonuclease Cas9		16E_02969	
CRISPR-associated endonuclease Cas9			Lm_XYSN_02735
CRISPR-associated endonuclease Cas9		16E_02998	
ESAT-6 secretion machinery protein EssC		16E_00100	
hypothetical protein		16E_00368	
hypothetical protein		16E_00184	
Lactose transport system permease protein LacF		16E_00905	
UDP-N-acetylmuramoylalanine--D-glutamate ligase		16E_02995	
hypothetical protein			Lm_XYSN_01890
Internalin B		16E_00489	
Phosphomevalonate kinase		16E_00022	
PTS system beta-glucoside-specific EIIBCA component		16E_00060	
30S ribosomal protein S18		16E_02973	

Accessory gene regulator protein B		16E_02975	
hypothetical protein		16E_00198	
hypothetical protein	15LG_00107		
hypothetical protein	15LG_00126		
Alpha-xylosidase		16E_00230	
hypothetical protein	15LG_00286		
hypothetical protein			Lm_XYSN_00499
hypothetical protein	15LG_00389		
hypothetical protein		16E_00548	
Serine/threonine-protein kinase PknB	15LG_00626		

Supplementary Table 9: Details of the 31 regions of differences (RODs) observed in the *L. monocytogenes* XYSN as compared to EGD-e. Yellow highlights indicates RODs detected in *L. ivanovii*. Blue highlights indicates RODs that were exclusive to *L. innocua*.

#RODs	Start	End	Length	encodes-for	BLAST indicates this region is present in
1	21897	23868	1972	Lipase, HP	*L. welshimeri* , *L. monocytogenes* lineage-III
2	25166	32328	7163	peptidoglycan binding protein, Plasmid pRiA4b ORF-3-like protein	*L. welshimeri*, *L. seeligeri* and *L. monocytogenes*
3	39463	44440	4978	microcin C7 resistance protein, ABC transporter, HP	*L. ivanovii* and *L. monocytogenes*
4	83114	97980	14867	TVII secretion protein EssC, LxG, HP, Ugp operon	EssC and LxG unique to XYSN, Ugp operon only in *L. ivanovii*
5	101026	110607	9582	transposase, integrase, HP, LXG-domain proteins	partially in *L. ivanovii*
6	126161	132161	6001	peptidoglycan binding protein	*L. seeligeri* and *L. monocytogenes*
7	142756	149455	6700	TVII secretion effector, LxG, and IS	except MGE, segment occurs in other *L. monocytogenes* strains
8	314558	319611	5054	ABC transporter, HP	*L. monocytogenes*
9	457341	479954	22614	wapA operon, transposases	wapA operon in *L. monocytogenes*
10	507983	512322	4340	TVII secretion effector, LXG protein, colicin, HP	*L. monocytogenes*
11	551449	552060	612	Type I restriction modification	*L. monocytogenes*
12	745082	751261	6180	peptidoglycan binding protein	*L. welshimeri*, *L. seeligeri* and *L. monocytogenes*
13	770378	774943	4566	Menaquinone biosynthesis operon (?)	*L. ivanovii*
14	790506	795578	5073	HP, transposase, antibiotic resistance protein vanZ	*L. welshimeri*, *L. ivanovii* and *L. seeligeri*
15	826605	839583	12979	oxidoreductase, RNA polymerase, peptidoglycan binding protein	*L. monocytogenes*
16	841193	848528	7336	peptidoglycan binding protein	*L. welshimeri* and *L. monocytogenes*
17	1108146	1126313	18168	homologues to rml operon/WTA	*L. monocytogenes*
18	1132807	1135213	2407	TVII secretion effector, LXG domain protein	*L. monocytogenes*
19	1193149	1194253	1105	membrane protein	*L. monocytogenes*
20	1245812	1253371	7560	Sphingomylinase and i-inlFE, IS256 transposase	*L. ivanovii*
21	1431313	1435095	3783	IS256 family transposase, integrase, HP	IS256 transposase from *L. weihenstephanensis* and HP from *L. ivanovii*
22	1707242	1716961	9720	CAAX amino terminal protein, integrase, HP	*L. innocua*, *L. monocytogenes*
23	1773357	1779404	6048	Beta lactamase, excinuclease	*L. innocua*
24	2211006	2212929	1924	NAD(P)H dehydrogenase	Unique (although low homology to few of *L. monocytogenes* strains)
25	2504847	2505908	1062	Cell surface protein (Leucine-rich domain)	several *L. monocytogenes*
26	2535962	2544430	8469	Leucine-rich repeat, IS256 family transposase and HP, integrase	Leucine-rich repeats: *L. ivanovii*, HP: *L. monocytogenes*
27	2663252	2666011	2760	Leucine-rich repeat-LPxTG protein	Low homology *L. monocytogenes*
27	2678257	2717469	39213	prophage, internalin C, HP	Prophage: low homology *L. innocua*, internalin C: *L. ivanovii*, HP: *L. monocytogenes*
29	2729551	2737952	8402	Type II crispr, IS5/IS1182 family transposase	*L. monocytogenes*

30	2911964	2915445	3482	Fic protein, multi drug transporter	*L. ivanovii* and *L. monocytogenes*
31	2927927	2935695	7769	Cobalt transporter operon and other proteins	*L. ivanovii*

Supplementary Table 10: Details of the 90 genes encoding putative transposases in the chromosome of *L. monocytogenes* XYSN.

5' location	3' location	length	Product	IS-classification (transposase family)	BLAST against nucleotide database indicates these transposases can be also found in
103788	105032	1245	Transposase	IS256	*L. newyorkensis* and *L. weihenstephanensis*
137069	137374	306	Transposase	IS3	*L. monocytogenes*
137371	138150	780	Transposase		
147286	147843	558	Transposase	IS3	*Enterococcus* spp.
147885	148601	717	transposase		
180455	181699	1245	transposase	IS256	*L. newyorkensis* and *L. weihenstephanensis*
195118	196515	1398	transposase DDE domain protein	IS5/IS1182	*L. ivanovii*, some *L. monocytogenes*, *Bacillus* spp.
207318	207977	660	transposase	IS3	*L. monocytogenes*
207974	208279	306	transposase		
266685	267242	558	transposase	IS3	*Enterococcus* spp.
267284	267781	498	transposase		
375975	376691	717	transposase	IS3	*Enterococcus* spp.
376733	377290	558	transposase		
407420	407587	168	transposase	LxG domain	*L. monocytogenes*
407682	408926	1245	transposase	IS256	*L. newyorkensis* and *L. weihenstephanensis*
474324	475568	1245	transposase	IS256	*L. newyorkensis* and *L. weihenstephanensis*
475666	475929	264	transposase (InsE family)	InsE family	
476994	477887	894	transposase		*L. ivanovii*
477851	478129	279	transposase		
478845	479219	375	transposase	HTH superfamily protein	
479625	479954	330	transposase	HTH superfamily protein	
489339	490664	1326	transposase	IS3	*Bacillus* spp., *L. newyorkensis*
544082	544885	804	transposase	IS3	*Enterococcus* spp.
544927	545484	558	transposase		
593980	594537	558	transposase	IS3	*Enterococcus* spp.
594579	595382	804	transposase		
629918	630475	558	transposase	IS3	*Enterococcus* spp.
630517	631320	804	transposase		
655410	656807	1398	transposase DDE domain protein	IS5/IS1182	*L. ivanovii*, some *L. monocytogenes*, *Bacillus* spp.

693798	694082	285	transposase		
702892	704289	1398	transposase DDE domain protein	IS5/IS1182	*L. ivanovii*, some *L. monocytogenes*, *Bacillus* spp.
791847	792149	303	transposase	IS256	*L. newyorkensis* and *L. weihenstephanensis*
831889	833133	1245	transposase	IS256	*L. newyorkensis* and *L. weihenstephanensis*
871489	871773	285	transposase		
945733	947130	1398	transposase DDE domain protein	IS5/IS1182	*L. ivanovii*, some *L. monocytogenes*, *Bacillus* spp.
951618	953015	1398	transposase DDE domain protein	IS5/IS1182	*L. ivanovii*, some *L. monocytogenes*, *Bacillus* spp.
967894	969291	1398	transposase DDE domain protein	IS5/IS1182	*L. ivanovii*, some *L. monocytogenes*, *Bacillus* spp.
1097652	1098008	357	transposase	IS256	*L. newyorkensis* and *L. weihenstephanensis*
1098018	1098896	879	transposase		
1138535	1139932	1398	transposase DDE domain protein	IS5/IS1182	*L. ivanovii*, some *L. monocytogenes*, *Bacillus* spp.
1146095	1147492	1398	transposase DDE domain protein	IS5/IS1182	*L. ivanovii*, some *L. monocytogenes*, *Bacillus* spp.
1249437	1250681	1245	transposase	IS256	*L. newyorkensis* and *L. weihenstephanensis*
1250801	1251067	267	transposase		*L. ivanovii*
1268874	1270199	1326	transposase	IS3	*Bacillus* spp., *L. newyorkensis*
1300425	1301669	1245	transposase	IS256	*L. newyorkensis* and *L. weihenstephanensis*
1301978	1302865	888	transposase	IS3	*L. fleischmannii*
1302877	1303572	696	transposase		
1347666	1349063	1398	transposase DDE domain protein	IS5/IS1182	*L. ivanovii*, some *L. monocytogenes*, *Bacillus* spp.
1392430	1393827	1398	transposase DDE domain protein	IS5/IS1182	*L. ivanovii*, some *L. monocytogenes*, *Bacillus* spp.
1431313	1432557	1245	transposase	IS256	*L. newyorkensis* and *L. weihenstephanensis*
1519181	1520578	1398	transposase DDE domain protein	IS5/IS1182	*L. ivanovii*, some *L. monocytogenes*, *Bacillus* spp.
1534704	1535591	888	transposase	IS3	*L. fleischmannii*
1535603	1536298	696	transposase		
1552182	1553579	1398	transposase DDE domain protein	IS5/IS1182	*L. ivanovii*, some *L. monocytogenes*, *Bacillus* spp.
1582112	1583509	1398	transposase DDE domain protein	IS5/IS1182	*L. ivanovii*, some *L. monocytogenes*, *Bacillus* spp.
1628197	1629000	804	transposase	IS3	*Enterococcus* spp.
1629042	1629599	558	transposase		
1641886	1643130	1245	transposase	IS256	*L. newyorkensis* and *L. weihenstephanensis*
1667416	1668813	1398	transposase DDE domain protein	IS5/IS1182	*L. ivanovii*, some *L. monocytogenes*, *Bacillus* spp.
1762079	1762882	804	transposase	IS3	*Enterococcus* spp.
1762924	1763481	558	transposase		
1809869	1810672	804	transposase	IS3	*Enterococcus* spp.
1810714	1811271	558	transposase		

1871317	1872714	1398	transposase DDE domain protein	IS5/IS1182	*L. ivanovii*, some *L. monocytogenes*, *Bacillus* spp.
1875526	1876923	1398	transposase DDE domain protein	IS5/IS1182	*L. ivanovii*, some *L. monocytogenes*, *Bacillus* spp.
1877072	1878469	1398	transposase DDE domain protein	IS5/IS1182	*L. ivanovii*, some *L. monocytogenes*, *Bacillus* spp.
1926400	1927797	1398	transposase DDE domain protein	IS5/IS1182	*L. ivanovii*, some *L. monocytogenes*, *Bacillus* spp.
2035488	2036291	804	transposase	IS3	*Enterococcus* spp.
2036333	2036890	558	transposase		
2045153	2046550	1398	transposase DDE domain protein	IS5/IS1182	*L. ivanovii*, some *L. monocytogenes*, *Bacillus* spp.
2065256	2066143	888	transposase	IS3	*L. fleischmannii*
2066155	2066850	696	transposase		
2070994	2072391	1398	transposase DDE domain protein	IS5/IS1182	*L. ivanovii*, some *L. monocytogenes*, *Bacillus* spp.
2090441	2091838	1398	transposase DDE domain protein	IS5/IS1182	*L. ivanovii*, some *L. monocytogenes*, *Bacillus* spp.
2167416	2168813	1398	transposase DDE domain protein	IS5/IS1182	*L. ivanovii*, some *L. monocytogenes*, *Bacillus* spp.
2178227	2178592	366	transposase	HTH superfamily protein	
2178744	2179988	1245	transposase	IS256	*L. newyorkensis* and *L. weihenstephanensis*
2179989	2180495	507	transposase		
2180507	2181202	696	transposase		
2192511	2193908	1398	transposase DDE domain protein	IS5/IS1182	*L. ivanovii*, some *L. monocytogenes*, *Bacillus* spp.
2244879	2246276	1398	transposase DDE domain protein	IS5/IS1182	*L. ivanovii*, some *L. monocytogenes*, *Bacillus* spp.
2270509	2271753	1245	transposase	IS256	*L. newyorkensis* and *L. weihenstephanensis*
2451719	2452897	1179	transposase DDE domain protein	IS5/IS1182	*L. ivanovii*, some *L. monocytogenes*, *Bacillus* spp.
2452929	2454254	1326	transposase	IS3	*Bacillus* spp., *L. newyorkensis*
2479598	2480995	1398	transposase DDE domain protein	IS5/IS1182	*L. ivanovii*, some *L. monocytogenes*, *Bacillus* spp.
2498728	2499972	1245	transposase	IS256	*L. newyorkensis* and *L. weihenstephanensis*
2539378	2540622	1245	transposase	IS256	*L. newyorkensis* and *L. weihenstephanensis*
2735845	2737242	1398	transposase DDE domain protein	IS5/IS1182	*L. ivanovii*, some *L. monocytogenes*, *Bacillus* spp.
2835844	2837169	1326	transposase	IS3	*Bacillus* spp., *L. newyorkensis*
2985439	2986836	1398	transposase DDE domain protein	IS5/IS1182	*L. ivanovii*, some *L. monocytogenes*, *Bacillus* spp.

References:

1. Doumith, M., Buchrieser, C., Glaser, P., Jacquet, C. & Martin, P. Differentiation of the Major *Listeria monocytogenes* Serovars by Multiplex PCR Differentiation of the Major *Listeria monocytogenes* Serovars by Multiplex PCR. *J. Clin. Microbiol.* **42,** 3819–3822 (2004).

2. Shen, Y. *et al.* Structural and functional diversity in *Listeria* cell wall teichoic acids. *J. Biol. Chem.* jbc.M117.813964 **292**(43), 17832-17844 (2017). doi:10.1074/jbc.M117.813964

3. Seeliger, H. P. R. & Höhne, K. Chapter II Serotyping of *Listeria monocytogenes* and related Species. *Methods Microbiol.* **13,** 31–49 (1979).

4. Sullivan, M. J., Petty, N. K. & Beatson, S. A. Easyfig: a genome comparison visualizer. *Bioinformatics* **27,** 1009–1010 (2011).

5. Sahl, J. W., Caporaso, J. G., Rasko, D. A. & Keim, P. The large-scale blast score ratio (LS-BSR) pipeline: a method to rapidly compare genetic content between bacterial genomes. *PeerJ* **2,** e332 (2014).

6. Hadfield, J. *et al.* Phandango: an interactive viewer for bacterial population genomics. *Bioinformatics* **34,** 292–293 (2018).

7. Chen, L., Zheng, D., Liu, B., Yang, J. & Jin, Q. VFDB 2016: Hierarchical and refined dataset for big data analysis - 10 years on. *Nucleic Acids Res.* **44,** D694-D697 (2016).

8. Page, A. J. *et al.* Roary: rapid large-scale prokaryote pan genome analysis. *Bioinformatics* **31,** 3691-3693 (2015).
