# Supplementary material for: A hybrid sub-lineage of Listeria monocytogenes comprising hypervirulent isolates
Source: Nat Commun. 2019 Sep 30;10:4283. doi: 10.1038/s41467-019-12072-1 (PMC6768887; doi:10.1038/s41467-019-12072-1)
Supplement: Supplementary file 2 — Description of Additional Supplementary Files [file 41467_2019_12072_MOESM2_ESM.docx]

**Description of Additional Supplementary Files**

**File Name: Supplementary Movie 1**

**Description:** Adhesion of XYSN to Caco-2 BBe cells. The relative localization of bacteria to Caco-2 BBe cells are visualised using cell tomography. The longitudinal coordinates (z) represents the height of cells, “Z=0 μm’’ represents the surface of the cell. The bacteria XYSN were present at the height of Z=0 μm, indicating bacteria adhered the surface of the cell.

**File Name: Supplementary Movie 2**

**Description:** Adhesion of Δ1095 to Caco-2 BBe cells. The relative localization of bacteria to Caco-2 BBe cells are visualised using cell tomography. The longitudinal coordinates (z) represents the height of cells, “Z=0 μm’’ represents the surface of the cell. The bacteria Δ1095 were present at the height of Z=0 μm, indicating bacteria adhered the surface of the cell.

**File Name: Supplementary Movie 3**

**Description:** Adhesion of Δ1095::1095 to Caco-2 BBe cells. The relative localization of bacteria to Caco-2 BBe cells are visualised using cell tomography. The longitudinal coordinates (z) represents the height of cells, “Z=0 μm’’ represents the surface of the cell. The bacteria Δ1095::1095 were present at the height of Z=0 μm, indicating bacteria adhered the surface of the cell.

**File Name: Supplementary Movie 4**

**Description:** Invasion of XYSN to Caco-2 BBe cells. The relative localization of bacteria to Caco-2 BBe cells were visualised using cell tomography. The longitudinal coordinates (z) represents the height of cells. The rod-shape GFP-labelled bacteria XYSN were present at the height of Z=3 μm, indicating bacterial entry into cells.

**File Name: Supplementary Movie 5**

**Description:** Invasion of Δ1095 to Caco-2 BBe cells. The relative localization of bacteria to Caco-2 BBe cells were visualised using cell tomography. The longitudinal coordinates (z) represents the height of cells. The rod-shape GFP-labelled bacteria XYSN were present at the height of Z=3 μm, indicating bacterial entry into cells. The rod-shape GFP-labelled bacteria Δ1095 were visualized Z=0 μm, and indicate the inability of bacteria to invade the cell.

**File Name: Supplementary Movie 6**

**Description:** Invasion of Δ1095::1095 to Caco-2 BBe cells. The relative localization of bacteria to Caco-2 BBe cells were visualised using cell tomography. The longitudinal coordinates (z) represents the height of cells. The rod-shape GFP-labelled bacteria XYSN were present at the height of Z=3 μm, indicating bacterial entry into cells. The rod-shape GFP-labelled bacteria Δ1095::1095 were visualised at the height of Z=4 μm, indicating invasion of bacteria into the cell.
