# Supplementary material for: A hybrid sub-lineage of Listeria monocytogenes comprising hypervirulent isolates
Source: Nat Commun. 2019 Sep 30;10:4283. doi: 10.1038/s41467-019-12072-1 (PMC6768887; doi:10.1038/s41467-019-12072-1)
Supplement: Supplementary file 9 — Reporting Summary [file 41467_2019_12072_MOESM9_ESM.pdf]

natureresearch

Corresponding author(s): Xin'an Jiao

Last updated by author(s): Jul 18, 2019

Reporting Summary

Nature Research wishes to improve the reproducibility of the work that we publish. This form provides structure for consistency and transparency in reporting. For further information on Nature Research policies, see Authors & Referees and the Editorial Policy Checklist.

Statistics

For all statistical analyses, confirm that the following items are present in the figure legend, table legend, main text, or Methods section.

n/a	Confirmed	
☐	☒	The exact sample size (*n*) for each experimental group/condition, given as a discrete number and unit of measurement
☐	☒	A statement on whether measurements were taken from distinct samples or whether the same sample was measured repeatedly
☐	☒	The statistical test(s) used AND whether they are one- or two-sided<br>*Only common tests should be described solely by name; describe more complex techniques in the Methods section.*
☒	☐	A description of all covariates tested
☒	☐	A description of any assumptions or corrections, such as tests of normality and adjustment for multiple comparisons
☒	☐	A full description of the statistical parameters including central tendency (e.g. means) or other basic estimates (e.g. regression coefficient) AND variation (e.g. standard deviation) or associated estimates of uncertainty (e.g. confidence intervals)
☒	☐	For null hypothesis testing, the test statistic (e.g. *F*, *t*, *r*) with confidence intervals, effect sizes, degrees of freedom and *P* value noted<br>*Give P values as exact values whenever suitable.*
☒	☐	For Bayesian analysis, information on the choice of priors and Markov chain Monte Carlo settings
☒	☐	For hierarchical and complex designs, identification of the appropriate level for tests and full reporting of outcomes
☒	☐	Estimates of effect sizes (e.g. Cohen's *d*, Pearson's *r*), indicating how they were calculated

Our web collection on statistics for biologists contains articles on many of the points above.

Software and code

Policy information about availability of computer code

Data collection	All the algorithms or software used in the manuscript are previously published and the same are cited in the manuscript
Data analysis	*Provide a description of all commercial, open source and custom code used to analyse the data in this study, specifying the version used OR state that no software was used.*

For manuscripts utilizing custom algorithms or software that are central to the research but not yet described in published literature, software must be made available to editors/reviewers. We strongly encourage code deposition in a community repository (e.g. GitHub). See the Nature Research guidelines for submitting code & software for further information.

Data

Policy information about availability of data

All manuscripts must include a data availability statement. This statement should provide the following information, where applicable:

- Accession codes, unique identifiers, or web links for publicly available datasets
- A list of figures that have associated raw data
- A description of any restrictions on data availability

Provide your data availability statement here.

Field-specific reporting

Please select the one below that is the best fit for your research. If you are not sure, read the appropriate sections before making your selection.

☒ Life sciences ☐ Behavioural & social sciences ☐ Ecological, evolutionary & environmental sciences

October 2018

Life sciences study design

All studies must disclose on these points even when the disclosure is negative.

Sample size	The sample size of animals experiments depends on the results of preliminary data and compliance the statistical analysis. For example, the survival experiment with guinea pigs was chosen 10 animals per group, because the variety between guinea pigs exists via oral infection.
Data exclusions	No data were excluded.
Replication	All the animal experiments were replicated.
Randomization	For bacterial loads in each organs, i.e., liver samples, 6 samples from 6 mice, other samples in each group are following this principle.
Blinding	We were blinded to group allocation during data collection.

Reporting for specific materials, systems and methods

We require information from authors about some types of materials, experimental systems and methods used in many studies. Here, indicate whether each material, system or method listed is relevant to your study. If you are not sure if a list item applies to your research, read the appropriate section before selecting a response.

Materials & experimental systems

n/a	Involved in the study
☐	☒ Antibodies
☐	☒ Eukaryotic cell lines
☒	☐ Palaeontology
☐	☒ Animals and other organisms
☒	☐ Human research participants
☒	☐ Clinical data

Methods

n/a	Involved in the study
☒	☐ ChIP-seq
☒	☐ Flow cytometry
☒	☐ MRI-based neuroimaging

Antibodies

Antibodies used	Anti-Ami antiserum (R5, kind gift from Pascale Cossart, Institut Pasteur), Anti-InlA (CSB-PA758331HA01AAD, CUSABIO, China), Anti-LLO (Monoclonal antibody, clone 3B6, prepared by our laboratory), Anti-ActA (Monoclonal antibody, clone 6F5, prepared by our laboratory), Anti-mouse IgG (401215, Calbiochemand, Germany), Anti-rabbit IgG (BS13278, Bioworld, United States), Listeria O-antiserum 4 (223011, BD, United States), Anti-mouse IgG conjugated with Alexa Fluor 488 (AB150113, abcam, England), Anti-rabbit IgG conjugated with Alexa Fluor 488 (AB150077, abcam, England).
Validation	Rabbit polyclonal anti-Ami antiserum at a 1:5000 dilution, Rabbit polyclonal anti-InlA at a 1:2000 dilution, mouse monoclonal anti-LLO and anti-ActA at a 1:2000 dilution, HRP-labeled goat anti-mouse IgG and goat anti-rabbit IgG at a 1:8000 dilution, Listeria O-antiserum 4 at a 1:200 dilution, goat anti-mouse IgG conjugated with Alexa Fluor 488 and goat anti-rabbit IgG conjugated with Alexa Fluor 488 at a 1:200 dilution.

Eukaryotic cell lines

Policy information about cell lines

Cell line source(s)	ATCC CRL-2102 Caco-2 BBe.
Authentication	The cell line was authenticated.
Mycoplasma contamination	The cell line tested negative for mycoplasma contamination.
Commonly misidentified lines (See ICLAC register)	*Name any commonly misidentified cell lines used in the study and provide a rationale for their use.*

Animals and other organisms

Policy information about studies involving animals; ARRIVE guidelines recommended for reporting animal research

Laboratory animals	6-week-old female C57BL/6 mice, 6-week-old female BALB/c mice, 150 g-weight female guinea pigs

Wild animals	The study didn't involve wild animals.
Field-collected samples	The study didn't involve animals from this field.
Ethics oversight	Animal experiments were conducted in accordance with guidelines for the welfare and ethics of experimental animals. All animals were kept at the animal biosafety facilities according to procedures approved by the Institutional Animal Ethics Committee of Yangzhou University.

Note that full information on the approval of the study protocol must also be provided in the manuscript.
